# A systematic literature review of the clinical and socioeconomic burden of bronchiectasis

**DOI:** 10.1183/16000617.0049-2024

**Published:** 2024-09-04

**Authors:** James D. Chalmers, Marcus A. Mall, Pamela J. McShane, Kim G. Nielsen, Michal Shteinberg, Sean D. Sullivan, Sanjay H. Chotirmall

**Affiliations:** 1 University of Dundee, Dundee, UK; 2 Department of Pediatric Respiratory Medicine, Immunology and Critical Care Medicine, Charité – Universitätsmedizin Berlin, Berlin, Germany; 3 German Center for Lung Research (DZL), associated partner site, Berlin, Germany; 4 Berlin Institute of Health at Charité – Universitätsmedizin Berlin, Berlin, Germany; 5 University of Texas Health Science Center at Tyler, Tyler, TX, USA; 6 Paediatric Pulmonary Service, Department of Paediatrics and Adolescent Medicine, Copenhagen University Hospital, Rigshospitalet, Denmark; 7 Department of Clinical Medicine, University of Copenhagen, Copenhagen, Denmark; 8 European Reference Network on rare respiratory diseases (ERN-LUNG); 9 Lady Davis Carmel Medical Center, Haifa, Israel; 10 Technion – Israel Institute of Technology, The B. Rappaport Faculty of Medicine, Haifa, Israel; 11 CHOICE Institute, University of Washington, Seattle, WA, USA; 12 Lee Kong Chian School of Medicine, Nanyang Technological University, Singapore; 13 Department of Respiratory and Critical Care Medicine, Tan Tock Seng Hospital, Singapore

## Abstract

**Background:**

The overall burden of bronchiectasis on patients and healthcare systems has not been comprehensively described. Here, we present the findings of a systematic literature review that assessed the clinical and socioeconomic burden of bronchiectasis with subanalyses by aetiology (PROSPERO registration: CRD42023404162).

**Methods:**

Embase, MEDLINE and the Cochrane Library were searched for publications relating to bronchiectasis disease burden (December 2017–December 2022). Journal articles and congress abstracts reporting on observational studies, randomised controlled trials and registry studies were included. Editorials, narrative reviews and systematic literature reviews were included to identify primary studies. PRISMA guidelines were followed.

**Results:**

1585 unique publications were identified, of which 587 full texts were screened and 149 were included. A further 189 citations were included from reference lists of editorials and reviews, resulting in 338 total publications. Commonly reported symptoms and complications included dyspnoea, cough, wheezing, sputum production, haemoptysis and exacerbations. Disease severity across several indices and increased mortality compared with the general population was reported. Bronchiectasis impacted quality of life across several patient-reported outcomes, with patients experiencing fatigue, anxiety and depression. Healthcare resource utilisation was considerable and substantial medical costs related to hospitalisations, treatments and emergency department and outpatient visits were accrued. Indirect costs included sick pay and lost income.

**Conclusions:**

Bronchiectasis causes significant clinical and socioeconomic burden. Disease-modifying therapies that reduce symptoms, improve quality of life and reduce both healthcare resource utilisation and overall costs are needed. Further systematic analyses of specific aetiologies and paediatric disease may provide more insight into unmet therapeutic needs.

## Shareable abstract

**Bronchiectasis imposes a significant clinical and socioeconomic burden on patients, their families and employers, and on healthcare systems. Therapies that reduce symptoms, improve quality of life and reduce resource use and overall costs are needed.** https://bit.ly/4bPCHlp

## Introduction

Bronchiectasis is a heterogeneous chronic respiratory disease clinically characterised by chronic cough, excessive sputum production and recurrent pulmonary exacerbations [1], and radiologically characterised by the abnormal widening of the bronchi [2]. Bronchiectasis is associated with several genetic, autoimmune, airway and infectious disorders [3]. Regardless of the underlying cause, the defining features of bronchiectasis are chronic airway inflammation and infection, regionally impaired mucociliary clearance, mucus hypersecretion and mucus obstruction, as well as progressive structural lung damage [4, 5]. These features perpetuate one another in a “vicious vortex” leading to a decline in lung function, pulmonary exacerbations and associated morbidity, mortality and worsened quality of life [4, 5]. Bronchiectasis can be further categorised into several infective and inflammatory endotypes and is associated with multiple comorbidities and underlying aetiologies [6].

Bronchiectasis has been described as an emerging global epidemic [7], with prevalence and incidence rates increasing worldwide [8–12]. The prevalence of bronchiectasis, as well as of the individual aetiologies, varies widely across geographic regions [13]. In Europe, the reported prevalence ranges from 39.1 (females) and 33.3 (males) cases per 100 000 inhabitants in Spain and 68 (females) and 65 (males) cases per 100 000 inhabitants in Germany, to as high as 566 cases (females) and 486 cases (males) per 100 000 inhabitants in the UK [10–12]. In the US, the average overall prevalence was reported to be 139 cases per 100 000 [14], in Israel, the prevalence was reported to be 234 cases per 100 000 [15], and in China the prevalence was reported to be 174 per 100 000 [8]. Studies show that bronchiectasis prevalence increases with age [14]. This may increase the socioeconomic impact of bronchiectasis on countries with disproportionately higher number of older citizens. Large registry studies in patients with bronchiectasis have been published from the US (Bronchiectasis Research Registry) [16], Europe and Israel (European Multicentre Bronchiectasis Audit and Research Collaboration (EMBARC)); the largest and most comprehensive report available to date) [17], India (EMBARC-India) [18, 19], Korea (Korean Multicentre Bronchiectasis Audit and Research Collaboration) [20] and Australia (Australian Bronchiectasis Registry) [21].

Although there are currently no approved disease-modifying therapies for bronchiectasis [4], comprehensive clinical care recommendations for the management of patients with bronchiectasis have been published [22, 23]. However, the burden that bronchiectasis imposes on patients and their families, as well as on healthcare systems, payers and employers, remains poorly understood. No review to date has used a systematic method to evaluate the overall disease burden of bronchiectasis. This is the first systematic literature review aimed at investigating and synthesising the clinical and socioeconomic burden of bronchiectasis. A better understanding of the overarching burden of bronchiectasis, both overall and by individual aetiologies and associated diseases, will highlight the need for new therapies and assist healthcare systems in planning care and required resources.

## Methods

The protocol of this systematic review was registered on PROSPERO (reference number: CRD42023404162).

### Search strategy

This systematic literature review was conducted according to the Preferred Reporting Items for Systematic Review and Meta-Analyses (PRISMA) guidelines [24]. Embase, MEDLINE and the Cochrane Library were searched for studies related to the clinical and socioeconomic burden of bronchiectasis (noncystic fibrosis bronchiectasis (NCFBE) and cystic fibrosis bronchiectasis (CFBE)) using the search terms available in supplementary table S1. Articles written in English and published over a 5-year period (December 2017–December 2022) were included.

### Selection criteria

The following article types reporting on prospective and retrospective observational studies, registry studies and randomised controlled trials (only baseline data extracted) were included: journal articles, preprints, research letters, conference proceedings, conference papers, conference abstracts, meeting abstracts and meeting posters. Reviews, literature reviews, systematic reviews and meta-analyses, as well as editorials, commentaries, letters and letters to the editor, were included for the purpose of identifying primary studies. A manual search of references cited in selected articles was performed and references were only included if they were published within the 5 years prior to the primary article being published.

### Screening and data extraction

A reviewer screened all titles and abstracts to identify publications for full-text review. These publications then underwent full-text screening by the same reviewer for potential inclusion. A second reviewer independently verified the results of both the title/abstract screen and the full-text screen. Any discrepancies were resolved by a third independent reviewer. Data relating to aetiology, symptoms, disease severity, exacerbations, lung function, infection, comorbidities, patient-reported outcomes (PROs), exercise capacity, mortality, impact on family and caregivers, healthcare resource utilisation (HCRU), treatment burden, medical costs, and indirect impacts and costs, as well as data relating to the patient population, study design, sample size and country/countries of origin, were extracted from the final set of publications into a standardised Excel spreadsheet by one reviewer. Studies were grouped based on the burden measure, and aggregate data (range of reported values) were summarised in table or figure format. For the economic burden section, costs extracted from studies reporting in currencies other than the euros were converted to euros based on the average exchange rate for the year in which the study was conducted.

Data from patients with specific bronchiectasis aetiologies and in children (age limits varied from study to study and included upper age limits of 15, 18, 19 and 20 years) were reported separately, where available. As literature relating to NCFBE and CFBE is generally distinct, any data related to CFBE are reported separately in the tables and text. We conducted subanalyses of key disease burden indicators, in which we extracted data from multicentre studies or those with a sample size >1000 subjects, to try to identify estimates from the most representative datasets. These data from larger and multicentre studies are reported in square brackets in tables 1–3 and supplementary tables S2–S7, where available.

**TABLE 1:** Prevalence and severity of bronchiectasis symptoms overall, in children, during exacerbations and in individual bronchiectasis aetiologies

Symptom	Prevalence overall (range of %)	Prevalence in children (range of %)	Prevalence during exacerbation**^a^** **or in frequent exacerbators (range of %)**	Prevalence by aetiology (% or range of %)	Number of studies
**Dyspnoea prevalence** **^b^**	7.1–78.1 (26.1–33.5)	19.7–81.3	83.9–87.2^c^ 30.0–32.4^d^	–	13
**Dyspnoea severity**					
MRC score^e^ (mean or median)	1.8–3.0^f^ 0.0–3.0^g^	–	0.0–2.0^h^	Post-TB (2.8), idiopathic (2.4), other aetiologies (2.3)^i^	20
MRC score: 0	9.0	–	–	CF (58.3)^j^	2
MRC score: 1	22.4–37.5	–	–	CF (30.3)^j^	3
MRC score: 2	19.9–43.3	–	–	CF (8.3)^j^	3
MRC score: 3	20.4–22.4	–	–	CF (3.0)^j^	3
MRC score: 4	3.0–14.3	–	–	–	4
MRC score: 5	5.0–7.9		–	–	2
mMRC score^k^ (mean or median)	1.1–2.3^f^ 1.0–2.0^g^	–	1.8^l^–2.2^m^	Post-TB (2.0), post-infectious (2.0), asthma (2.0), COPD (2.0), other aetiologies (2.0), idiopathic (1.0), ABPA (1.0)^n^	28
				Idiopathic or post-infectious (2.4)^o^	
mMRC score: 0	7.7–65.7	–	–	–	6
mMRC score: 1	18.2–51.6	–	–	–	6
mMRC score: 2	3.6–34.2	–	–	–	6
mMRC score: 3	2.1–34.2	–	–	–	6
mMRC score: 4	0.7–9.1	–	–	–	5
**Cough**	24.0–98.5 (25.8–95.0)	41.9–98.3	78.7–84.9^c^ 94.5–95.0^d^	PCD (91.0)^p^, idiopathic (81.3), AATD (72.4), CVID (64.7)	32
**Wheezing**	15.0–65.3 (15.0–16.0)	1.5–52.5	–	PCD (70.5)^q^, idiopathic (29.1), AATD (25.9), CVID (33.3)	15
**Wheezing in previous 12 months**	–	30.9–47.7	–	–	3
**Sputum production**	22.0–92.7 (41.4–92.7)	6.1–77.9	85.1^r^ 43.2–62.5^d^	COPD (65.2)^s^, other aetiologies (50.8)	26
**Mucoid sputum**	9.1–46.4 (24.2–46.4)	–	–	CF (12.9)^j^	13
**Mucopurulent sputum**	17.7–80.1 (17.7–44.8)	–	–	CF (37.1)^j^	14
**Purulent sputum**	5.9–84.5 (5.9–84.5)	–	–	CF (40.9)^j^	14
**24-hour sputum volume (mL, mean or median)**	11.4–91.2^f^ 5.0–21.0^g^	–	–	COPD (20.0), post-TB (10.0), post-infectious (10.0), asthma (10.0), other aetiologies (10.0), idiopathic (5.0), ABPA (5.0)^n^	14
				CF (25.8)^j^	
**24-hour sputum weight (g, mean or median)**	10.8–41.8^f^ 15.4–21.1^g^	–	–	Idiopathic or post-infectious (15.5–37.1)^o^	8
**Haemoptysis**	2.4–63.5 (13.2–40.5)	6.7–16.3	8.0^u^ 8.5–13.9^c^ 17.6^v^	Post-TB (30.4)^t^, other aetiologies (11.8)	33
				Idiopathic (28.6), post-TB (25.4), ABPA (14.3), PCD (14.3), COPD (11.1), post-pneumonia (10.5)^w^	

All data are percentages except where mean or median are indicated. Parentheses in the “Prevalence overall” column indicate data from larger or multicentre studies only. All data from individual studies are available in the supplemental Excel file. ^a^: A study reported increased dyspnoea during an exacerbation in 77.5% of patients, increased cough during exacerbation in 85.0% of patients and increased sputum purulence and sputum volume in 83.4% and 72.2% of patients, respectively [25]. ^b^: Dyspnoea, only when active or exercising, was reported in two studies and ranged from 11.0 to 54.0% [16, 26]. ^c^: Single study in adults with noncystic fibrosis bronchiectasis (NCFBE) during a pneumonic or nonpneumonic exacerbation. No significant difference in the prevalence of dyspnoea (p=0.599), cough (p=0.356) or haemoptysis (p=0.422) was reported between pneumonic and nonpneumonic exacerbations [27]. ^d^: Single study in children with NCFBE during a virus-positive or virus-negative exacerbation. No significant difference in the prevalence of dyspnoea (p=0.81) or wet cough (p=0.93) was reported between virus-positive and -negative exacerbations; however, virus-negative exacerbations were significantly associated with sputum production (p=0.09) [28]. ^e^: The Medical Research Council (MRC) dyspnoea scale assesses the impact of breathlessness on an individual. MRC scores range from 1 to 5, with higher scores indicating more severe dyspnoea [29]. The grading is as follows: 1=not troubled by breathlessness except on strenuous exercise; 2=short of breath when hurrying on a level or when walking up a slight hill; 3=walks slower than most people on the level, stops after a mile or so, or stops after 15 min walking at own pace; 4=stops for breath after walking 100 yards, or after a few minutes on level ground; 5=too breathless to leave the house, or breathless when dressing/undressing. ^f^: Studies reporting mean. ^g^: Studies reporting median. ^h^: Median MRC dyspnoea score reported in a single study of patients admitted to hospital for an exacerbation [30]. In another study, patients hospitalised for an exacerbation during the 1-year follow-up period had significantly higher MRC dyspnoea scores compared with those who were not hospitalised for an exacerbation during the same follow-up period (p=0.004) [31]. ^i^: Patients with post-tuberculosis (TB) bronchiectasis had significantly higher MRC scores compared with idiopathic bronchiectasis and other aetiologies (not post-TB or idiopathic) (p<0.05 for all comparisons) [32]. ^j^: Study reporting in patients with cystic fibrosis (CF)-related bronchiectasis; no comparison with other aetiologies [33]. ^k^: The modified MRC (mMRC) dyspnoea scale is a slightly modified version of the MRC dyspnoea scale. mMRC scores range from 0 to 4, with higher scores indicating more severe dyspnoea [34]. Grading is as follows: 0=I only get breathless with strenuous exercise; 1=I get short of breath when hurrying on level ground or walking up a slight hill; 2=on level ground, I walk slower than people of the same age because of breathlessness, or have to stop for breath when walking at my own pace; 3=I stop for breath after walking about 100 yards or after a few minutes on level ground; 4=I am too breathless to leave the house or I am breathless when dressing. ^l^: In a single study, the mean mMRC dyspnoea score was significantly higher in frequent exacerbators (≥2 exacerbations per year) compared with nonfrequent exacerbators (p<0.001) [35]. ^m^: In a single study, the mean mMRC dyspnoea score was significantly higher in frequent exacerbators (≥2 exacerbations⋅year^−1^ or ≥1 hospitalisation⋅year^−1^) compared with nonfrequent exacerbators (p<0.0001) [36]. ^n^: No statistical analyses performed [19]. “Other” includes rheumatoid arthritis, primary ciliary dyskinesia (PCD), gastro-oesophageal reflux disease and nontuberculous mycobacteria infection. ^o^: Studies reporting in patients with idiopathic or post-infectious bronchiectasis; no comparison with other aetiologies [37, 38]. ^p^: Patients with PCD-related bronchiectasis had a significantly higher prevalence of cough compared with patients with alpha-1 antitrypsin deficiency (AATD)- and common variable immunodeficiency (CVID)-related bronchiectasis (p=0.012), but not idiopathic bronchiectasis [39]. ^q^: Patients with PCD-related bronchiectasis had a significantly higher prevalence of wheezing compared with idiopathic, AATD-associated and CVID-associated bronchiectasis (p<0.0001) [39]. ^r^: Single study in adults with NCFBE hospitalised for an exacerbation [40]. ^s^: A significantly greater proportion of patients with COPD-related bronchiectasis had daily expectoration compared with other aetiologies (p=0.001) [41]. ^t^: In patients admitted to hospital for an exacerbation, significantly more patients with post-TB bronchiectasis had haemoptysis compared with other aetiologies (p=0.006) [30]. ^u^: Single study in adults with NCFBE (PCD and primary immunodeficiency excluded) during an exacerbation [25]. ^v^: Single study in adults hospitalised for an exacerbation [30]. ^w^: In patients admitted to a tertiary care centre; no statistical analyses performed [42]. –: Symptoms for which data were not reported in children, during an exacerbation or in individual aetiologies; ABPA: allergic bronchopulmonary aspergillosis.

**TABLE 2:** Patient-reported outcome scores in patients with bronchiectasis overall and in individual bronchiectasis aetiologies

Patient-reported outcome		Score description	Score overall	Score by aetiology	Number of studies
**SGRQ**	Total score	Scores range from 0 to 100, with higher scores indicating more limitations	26.5–66.3^a^ (31.6–53.0)^a^ 29.0–59.0^b^ (29.0–59.0)^b^	Idiopathic or post-infectious (39.7–43.2)^c^	35
				NCFBE (27.4), CF (25.4)^d^	
				IBD (54.3), RA (46.8), ABPA (45.8), idiopathic or post-infectious (42.0), post-TB (41.8), other aetiologies (49.6)^e^	
	Symptoms score		32.3–64.0^a^ (56.8–61.0)^a^	Idiopathic or post-infectious (61.0)^b^	17
				NCFBE (46.3), CF (40.1)^d^	
	Activities score		25.9–59.7^a^	NCFBE (24.9), CF (27.0)^d^	14
	Impacts score		18.2–63.0^a^	NCFBE (22.6), CF (19.9)^d^	14
**QoL-B**	Respiratory symptoms score	QoL-B comprises eight domains; scores for each domain can range between 0 and 100, with higher scores indicating fewer symptoms or better functioning and HRQoL	23.2–91.4^a^ (54.0–57.8)^a^ 65.0–68.6^b^	IBD (54)^f^, other aetiologies (58)	21
				Immunodeficiency (63.5)^g^	
				Idiopathic or post-infectious (51.2)^c^	
				AATD (58.7)^h^	
				Post-TB (66.7), ABPA (66.7), post-infectious (63.0), asthma (63.0), idiopathic (59.3), COPD (57.5), other aetiologies (51.9)^i^	
	Physical functioning score		23.9–67.5^a^ (42.5–55.7)^a^ 53.5–83.0^b^	–	10
	Vitality score		22.8–63.8^a^ (48.7–53.1)^a^ 44.0–56.0^b^	–	9
	Role functioning score		33.3–82.3^a^ (59.2–64.7)^a^ 66.7–73.3^b^	–	11
	Emotional functioning score		31.3–87.9^a^ (76.9–87.9)^a^ 79–83.3^b^	–	10
	Social functioning score		30.8–72.4^a^ (51.3–65.0)^a^ 50.0–75.0^b^	–	11
	Treatment burden score		32.2–76.0^a^ (63.2–66.8)^a^ 56.0–78.0^b^	–	11
	Health perception score		35.5–68.8^a^ (42.4–47.3)^a^ 33.0–60.0^b^	–	11
**LCQ**	Total score	Each domain is scored from 1 to 7 and domain scores are added together to obtain a total score that can range from 3 to 21. Higher scores indicate a better quality of life	10.0–17.5^a^ (13.4–15.3)^a^ 11.0–16.6^b^ (11.0–14.0)^b^	CF (16.6)^j^	24
	Physical score		4.5–5.7^a^ (5.0)^a^	CF (5.3)^j^	8
	Psychological score		4.8–5.9^a^ (5.0)^a^	CF (5.3)^j^	8
	Social score		4.6–6.1^a^ (5.3)^a^	CF (5.7)^j^	8
**CAT**	CAT score	Scores range from 0 to 40, with higher scores denoting more severe impacts	14.3–21.2^a^ (14.8)^a^	Idiopathic or post-infectious (19.1–26.0)^c^	7
**BHQ**	BHQ score	Scores range from 0 to 100; higher scores indicate better health status	39.0–61.9^a^ (61.9)^a^ 53.5^b^	–	5
**HRQoL in children**					
PC-QoL^k^	PC-QoL score	Scores range from 1 (low quality of life) to 7 (high quality of life)	4.4–6.5^b^ (4.4–6.5)^b^	–	5
CC-QoL^k^	CC-QoL score	Scores range from 1 (low quality of life) to 7 (high quality of life)	6.5^b^ (6.5)^b^	–	1
PedsQL	Child-specific PedsQL score	Scores range from 0 to 100, with higher scores indicating better quality of life	Significantly lower in children with bronchiectasis compared with age-matched controls^l^	–	1
	Parent-proxy PedsQL score				
**Anxiety and depression**					
HADS	HADS-A score	Each subscore ranges from 0 to 21, with a higher score indicating more severe anxiety or depression^m^	4.4–7.0^a,n,o^ (4.4–4.9)^a^	NCFBE (7.0), CF (5.5)^d^	8
	HADS-D score		2.9–5.6^a,n,o^ (3.1–5.6)^a^	NCFBE (4.4), CF (3.6)^d^	8
PHQ-9	PHQ-9 score	Scores range from 0 to 27, with higher scores indicating more severe depression^p^	4.8–11.1^q^	–	1
**Fatigue**					
FSS	FSS score	Comprises nine categories (each are scored from 0 to 7); total score ranges from 9 to 63^r^	20.0–39.7^s^ 4.7–5.0^t^	–	3

Parentheses in the “Score by aetiology” column indicate data from larger or multicentre studies only. All data from individual studies are available in the supplemental Excel file. ^a^S: Studies reporting mean. ^b^: Studies reporting median. ^c^: Studies reporting in patients with idiopathic or post-infectious bronchiectasis; no comparison with other aetiologies [37, 44–47]. ^d^: No significant difference between patients with noncystic fibrosis bronchiectasis (NCFBE) and cystic fibrosis (CF)-related bronchiectasis (no p-value reported) [48]. ^e^: No significant difference between aetiologies (p=0.1) [49]. “Other” includes none of the aforementioned aetiologies. ^f^: Patients with inflammatory bowel disease (IBD)-related bronchiectasis had significantly worse Quality of Life–Bronchiectasis (QoL-B) respiratory symptom domain scores compared with patients with other aetiologies (p=0.02). The difference was greater when IBD-related bronchiectasis was compared with idiopathic bronchiectasis alone (mean difference: 5.6 points; p=0.001) [50]. ^g^: Patients with immunodeficiency-related bronchiectasis were found to have a significantly lower respiratory symptoms score according to QoL-B when compared with other aetiologies (mean difference: 3.5 points; p<0.0001) [51]. ^h^: tudy reporting in patients with alpha-1 antitrypsin deficiency (AATD)-related bronchiectasis; no comparison with other aetiologies [52]. ^i^: No statistical analyses were performed in this study [19]. “Other” includes rheumatoid arthritis (RA), primary ciliary dyskinesia, gastro-oesophageal reflux disease and nontuberculosis (TB) mycobacteria infection. ^j^: Study reported in patients with CF-related bronchiectasis; no comparison with other aetiologies [33]. ^k^: The parent cough-specific quality of life (PC-QoL) and the chronic cough-specific quality of life (CC-QoL) are instruments for assessing the impact of a child's chronic cough on quality of life; however, the PC-QoL is completed by the parent or caregiver of the child, whereas the CC-QoL is completed by the child themselves. ^l^: Single study comparing health-related quality of life (HRQoL) in children with bronchiectasis with age-matched controls with no diagnosis of a respiratory condition [53]. ^m^: Scores of ≥8 generally reflect the presence of anxiety or depression, with scores between 8–10, 11–14 and 15–21 reflecting mild, moderate and severe anxiety or depression, respectively. ^n^: In a study in which the presence of anxiety (Hospital Anxiety and Depression Scale–Depression (HADS)-A ≥8) or depression (HADS-D ≥8) was an inclusion criterion, the median HADS-A score was 10 (indicating mild anxiety) and the median HADS-D score was 11 (indicating moderate depression) [54]. ^o^: In one study, mean HADS-A and HADS-D scores were found to be significantly higher in patients with bronchiectasis (HADS-A: 7.0; HADS-D: 5.3) compared with matched healthy controls (HADS-A: 4.1; HADS-D: 3.5), indicating more severe anxiety and depression in patients with bronchiectasis (p<0.001 for both comparisons) [55]. ^p^: Scores of 0–4, 5–9, 10–14, 15–19 and 20–27 indicate minimal/no, mild, moderate, moderately severe and severe depression, respectively. ^q^: Mean Nine-question Patient Health Questionnaire (PHQ-9) scores were reported in a single study and ranged from 4.8 (minimal/no depression) in patients with mild bronchiectasis to 11.1 (moderate depression) in patients with severe bronchiectasis [56]. ^r^: Scores ≥36 indicate that a patient may be suffering from fatigue, with fatigue severity increasing with increasing score. ^s^: In a study in which the total score was reported, the maximum mean Fatigue Severity Scale (FSS) score presented was reported in bronchiectasis patients with depression (PHQ-9 ≥10); this was significantly higher than the mean FSS score in patients without depression (20.0) (p<0.001) [56]. ^t^: In studies in which the average score was reported (total score divided by 9) [57, 58]. –: Patient-reported outcomes for which data were not reported in individual aetiologies. ABPA: allergic bronchopulmonary aspergillosis; BHQ: Bronchiectasis Health Questionnaire; CAT: COPD Assessment Test; LCQ: Leicester Cough Questionnaire; PedsQL: Paediatric Quality of Life Inventory; SGRQ: St. George's Respiratory Questionnaire.

**TABLE 3:** Healthcare resource utilisation (HCRU) in patients with bronchiectasis overall and in individual bronchiectasis aetiologies

	HCRU overall	HCRU by aetiology	Number of studies
**Hospitalisations in the previous year (range of means or medians)**	0.2–1.8^a^ (0.4–1.2)^a^ 0.0^b^	Post-infectious (0.8), other aetiologies (0.5), COPD (0.4), PCD (0.4), idiopathic (0.3)^c^	11
		Post-TB (1.4), ABPA (1.3), idiopathic (1.2), post-pneumonia (1.2), immunodeficiency (1.2), rheumatic disease (0.7)^d^	
≥1 hospitalisation (range of %)	12.0–77.5 (12.0–61.0)		17
>1 hospitalisation (range of %)	7.0–59.9 (38.8)	COPD (54.3), other aetiologies (47.2), idiopathic (45.4), post-TB (42.9), asthma (38.9), post-infectious (34.8), ABPA (29.1)^e^	3
**Hospitalisations in the previous 2 years (range of means or medians)**	0.5–0.7^a^ 0.0–2.0^b^	–	4
≥1 hospitalisation (range of %)	10.0–39.0 (34.0–38.0)	PCD (48.3), CVID (44.4), AATD (19.3), idiopathic (18.2)^f^	5
**Hospitalisations per year (range of means)**	0.03–1.3^a^ (0.3–1.1)^a^	COPD (1.0–1.5)^g^	6
≥1 hospitalisation (range of %)	15.0–40.0 (32.0)	–	2
**Hospitalisations in first year of follow-up**		–	
≥1 hospitalisation (range of %)	0.0–42.0 (14.0–42.0)	–	4
**ED visits in the previous year (range of means)**	0.4–2.1^a^ (0.4)^a^	–	2
**ED visits per year (range of means)**	0.4–1.3^a^ (0.4–1.3)^a^	–	2
**Outpatient visits per year (range of means)**	6.8–21.0^a^ (6.8–21.0)^a^	–	2
**Length of stay (days) (range of means or medians)**	6.9–17.4^a^ (6.9–11.0)^a^ 4.0–47^b,h^ (4.0–12.0)^b^	–	18

Parentheses in the “HCRU overall” column indicate data from larger or multicentre studies only. All data from individual studies are available in the supplemental Excel file. ^a^: Studies reporting mean. ^b^: Studies reporting median. ^c^: No significant difference between aetiologies (no p-value given) [59]. ^d^: No significant difference between aetiologies (p=0.134) [60]. Rheumatic disease includes rheumatoid arthritis (RA), systemic lupus erythematosus, primary Sjögren's syndrome, vasculitis and ankylosing spondylitis. ^e^: No statistical analyses performed [19]. “Other” includes RA, primary ciliary dyskinesia (PCD), gastro-oesophageal reflux disease and non-tuberculosis (TB) mycobacteria infection. ^f^: A significantly higher proportion of patients with PCD-related bronchiectasis were hospitalised in the previous 2 years compared with patients with alpha-1 antitrypsin deficiency (AATD)-related and idiopathic bronchiectasis; additionally, significantly more patients with common variable immunodeficiency (CVID)-related bronchiectasis were hospitalised compared with idiopathic bronchiectasis (p<0.0001 for both comparisons) [39]. ^g^: Study reported in patients with COPD-related bronchiectasis of different severities; no comparison with other aetiologies [61]. ^h^: Maximum value reported in patients admitted to the intensive care unit with severe bronchiectasis [62]. –: Measures of HCRU for which data were not reported in individual aetiologies; ABPA: allergic bronchopulmonary aspergillosis; ED: emergency department.

Given the nature of the data included in this systematic literature review (that is, a broad range of patient clinical and socioeconomic characteristics rather than the outcome(s) of an intervention), in addition to the broad range of study types included, meta-analyses to statistically combine data of similar studies were not deemed appropriate and therefore not performed.

## Results

### Summary of included studies

A total of 1834 citations were retrieved from the Embase, MEDLINE and Cochrane Library databases, of which 1585 unique citations were identified. Abstract/title screening led to the inclusion of 587 citations for full-text screening. Following full-text screening, 149 primary citations and 110 literature reviews, systematic reviews and meta-analyses as well as editorials and letters to the editor remained. From the reference lists of these 110 citations, a further 189 primary citations were identified. These articles were only included if 1) the primary articles contained data relating to the burden of bronchiectasis and 2) the primary articles were published within the 5 years prior to the original article's publication date. In total, 338 publications were considered eligible and included in this review (supplementary figure S1). This included 279 journal articles, 46 congress abstracts and 13 letters to the editor or scientific/research letters. The results are summarised in the sections below. For the results from individual studies, including a description of the patient population, study design, sample size and country/countries of origin, please see the supplemental Excel file.

### Aetiology

The most frequently reported aetiologies included post-infectious, genetic (primary ciliary dyskinesia (PCD), alpha-1 antitrypsin deficiency (AATD) and cystic fibrosis (CF)), airway diseases (COPD and asthma), allergic bronchopulmonary aspergillosis (ABPA), aspiration and reflux-related, immunodeficiency and autoimmune aetiologies (supplementary figure S2). However, in up to 80.7% of adult cases and 53.3% of paediatric cases, the aetiology was not determined (referred to as “idiopathic bronchiectasis”) (supplementary figure S2). When limited to larger or multicentre studies, the frequency of idiopathic bronchiectasis ranged from 11.5 to 66.0% in adults and from 16.5 to 29.4% in children. Further details and additional aetiologies can be seen in the supplemental Excel file.

### Clinical burden

#### Symptom burden and severity

Commonly reported symptoms in patients with bronchiectasis included cough, sputum production, dyspnoea, wheezing and haemoptysis, with these symptoms more prevalent in adults compared with children (table 1). Other reported symptoms included chest discomfort, pain or tightness (both generally and during an exacerbation), fever and weight loss in both adults and children, and fatigue, tiredness or asthenia, appetite loss, and sweating in adults. In children, respiratory distress, hypoxia during an exacerbation, sneezing, nasal and ear discharge, thriving poorly including poor growth and weight loss, exercise intolerance, malaise, night sweats, abdominal pain, recurrent vomiting, and diarrhoea were reported (supplemental Excel file). Classic bronchiectasis symptoms such as sputum production (range of patients reporting sputum production across all studies: 22.0–92.7%) and cough (range of patients reporting cough across all studies: 24.0–98.5%) were not universally reported (table 1).

In a study comparing bronchiectasis (excluding CFBE) in different age groups (younger adults (18–65 years), older adults (66–75 years) and elderly adults (≥76 years) [63]), no significant differences across age groups were reported for the presence of cough (younger adults: 73.9%; older adults: 72.8%; elderly adults: 72.9%; p=0.90), sputum production (younger adults: 57.8%; older adults: 63.8%; elderly adults: 6.0%; p=0.16) or haemoptysis (younger adults: 16.5%; older adults: 19.3%; elderly adults: 16.3%; p=0.47).

#### Disease severity

Disease severity was reported according to several measures including the bronchiectasis severity index (BSI), the forced expiratory volume in 1 s (FEV_1_), Age, Chronic Colonisation, Extension, Dyspnoea (FACED) score and the Exacerbations-FACED (E-FACED) score, all of which are known to be associated with future exacerbations, hospitalisations and mortality (supplementary table S2 and the supplemental Excel file). Up to 78.7, 41.8 and 40.8% of patients with bronchiectasis reported severe disease according to the BSI, FACED score and E-FACED score, respectively (supplementary table S2). In most studies, severity scores were greater among people with bronchiectasis secondary to COPD or post-tuberculosis (TB) than idiopathic bronchiectasis (supplementary table S2). No data relating to disease severity were reported for CFBE specifically.

#### Exacerbations

**FIGURE 1:**
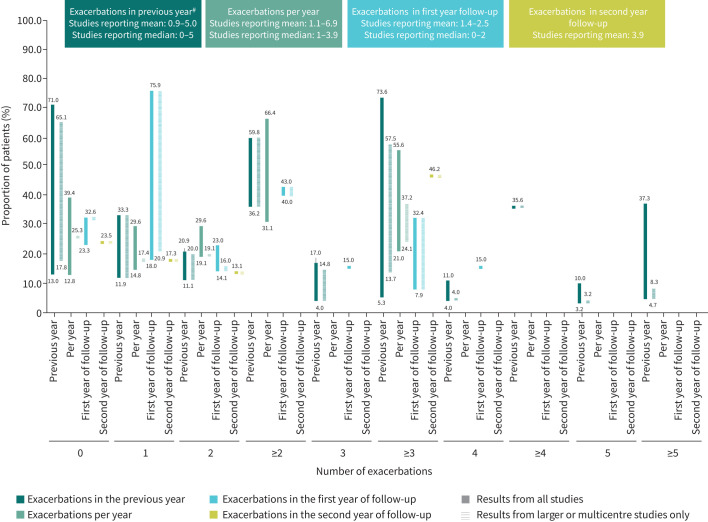
Range of bronchiectasis exacerbations in the previous year, per year and in the first and second years of follow-up. ^#^: Two studies reported significant differences in the number of exacerbations experienced in the previous year across individual aetiologies. Study 1 [90]: Patients with idiopathic bronchiectasis had significantly fewer exacerbations in the previous year compared with other aetiologies (primary ciliary dyskinesia (PCD), COPD and post-infectious) (p<0.021). Study 2 [33]: significant difference between post-tuberculosis (TB) bronchiectasis (mean: 2.8) and other aetiologies excluding idiopathic bronchiectasis (mean: 1.7) (p<0.05).

The number of exacerbations experienced by patients with bronchiectasis in the previous year, per year and during follow-up are presented in figure 1. For further details, please see the supplemental Excel file. Two studies reported exacerbation length in patients with bronchiectasis; this ranged from 11 to 16 days (both small studies; sample sizes of 191 and 32, respectively) [25, 64]. A study in children with NCFBE reported a median of one exacerbation in the previous year. Additionally, the same study reported that 31.1% of children with bronchiectasis experienced ≥3 exacerbations per year [65].

#### Lung function

Reduced lung function was reported across several different measures in adults and children with bronchiectasis overall, including FEV_1_ (absolute values and % predicted), forced vital capacity (FVC; absolute values and % pred) and lung clearance index (adults only) (supplementary table S3 and the supplemental Excel file). In most studies, lung function was lowest among people with post-TB bronchiectasis and bronchiectasis secondary to COPD or PCD (supplementary table S2). Additional measures of lung function are detailed in the supplemental Excel file. Lung clearance index, considered more sensitive than spirometry to early airway damage, was elevated in two studies in adults with bronchiectasis, with a range of 9.0–12.8 (normal: 6–7 or less) [66, 67].

In a study comparing bronchiectasis (people with CFBE excluded) in different age groups, elderly adults (≥76 years) had significantly lower FEV_1_ % pred (median: 67) compared with both younger (18–65 years; median: 78) and older adults (66–75 years; median: 75) (p<0.017 for both comparisons) [63]. FVC % pred was found to be significantly lower in elderly adults (mean: 65) compared with both younger adults (median: 78) and older adults (median: 75) (p<0.017 for both comparisons) [63].

#### Infections

Chronic infection with at least one pathogen was reported in 22.3–79.6% of patients with bronchiectasis, although each study defined chronic infection differently (number of studies: 20). When limited to larger or multicentre studies, chronic infection with at least one pathogen was reported in 10.7–54.5% of patients with bronchiectasis (number of studies: 12). In two studies in NCFBE, significant differences in the proportion of patients chronically infected with at least one pathogen were reported across aetiologies (p<0.001 for both studies) [68, 69]. Patients with post-infectious (other than TB) bronchiectasis (34.9%) [68] and patients with PCD-related bronchiectasis (68.3%) [69] had the highest prevalence of chronic infection.

The most commonly reported bacterial and fungal pathogens are shown in supplementary table S4. The two most common bacterial pathogens were *Pseudomonas* (*P*.) *aeruginosa* and *Haemophilus* (*H.*) *influenzae*. In several studies, more patients with PCD, TB and COPD as the aetiology of their bronchiectasis reported infection with *P. aeruginosa*. Additionally, in one study, significantly more children with CFBE had *P. aeruginosa* infection compared with children with NCFBE [70]. Further details and additional pathogens are reported in the supplemental Excel file.

Diversity of the sputum microbiome was assessed in two studies. In the first study in people with bronchiectasis (people with CFBE excluded), reduced microbiome alpha diversity (defined as the relative abundance of microbial species within a sample), particularly associated with *Pseudomonas* or Proteobacteria dominance, was associated with greater disease severity, increased frequency and severity of exacerbations, and a higher risk of mortality [71]. In the second study (unknown whether people with CFBE were excluded), a lower Shannon–Wiener diversity index (a measure of species diversity, with lower scores indicating lower diversity) score was associated with multiple markers of disease severity, including a higher BSI score (p=0.0003) and more frequent exacerbations (p=0.008) [72].

In a study comparing bronchiectasis (people with CFBE excluded) in different age groups (younger adults: 18–65 years; older adults: 66–75 years; elderly adults: ≥76 years) [63], chronic infection with *H. influenzae* was reported in 18.3% of younger adults, 12.8% of older adults and 8.8% of elderly adults, and chronic infection with *Streptococcus* (*Str.*) *pneumoniae* was reported in 5.3% of younger adults, 2.8% of older adults and 1.3% of elderly adults. For both of the above, the prevalence was significantly higher in younger adults compared with elderly adults (p<0.017 for both comparisons). However, no significant differences across age groups were reported for *P. aeruginosa*, *Moraxella catarrhalis* or *Staphylococcus* (*Sta*.) *aureus* chronic infection.

*P. aeruginosa* infection was significantly associated with reduced FEV_1_ [73], more severe disease [74], more frequent exacerbations [35, 49, 75, 76], increased hospital admissions, reduced quality of life based on St. George's Respiratory Questionnaire (SGRQ) and increased and 4-year mortality [49, 76]. Additionally, in a study reporting healthcare use and costs in the US between 2007–2013, healthcare costs and hospitalisation costs were found to be increased in patients infected with *P. aeruginosa* ($56 499 and $41 972 more than patients not infected with *P. aeruginosa*, respectively) [77]. In the same study, HCRU was also higher in patients infected with *P. aeruginosa* (fivefold increase in the number of hospitalisations and 84% more emergency department (ED) visits compared with patients not infected with *P. aeruginosa*) [77].

#### Comorbidities

The most frequently reported comorbidities included cardiovascular (including heart failure, cerebrovascular disease and hypertension), respiratory (including asthma, COPD and sinusitis), metabolic (including diabetes and dyslipidaemia), malignancy (including haematological and solid malignancies), bone and joint-related (including osteoporosis and rheumatological disease), neurological (including anxiety and depression), renal, hepatic, and gastrointestinal comorbidities (supplementary table S5). No data relating to comorbidities were reported for CFBE specifically. For further details and additional comorbidities, please see the supplemental Excel file.

In a study comparing bronchiectasis (people with CFBE excluded) in different age groups (younger adults: 18–65 years; older adults: 66–75 years; elderly adults: ≥76 years), younger adults had a significantly lower prevalence of diabetes compared with older adults, a significantly lower prevalence of stroke compared with elderly adults and a significantly lower prevalence of heart failure, solid tumours and renal failure compared with both older and elderly adults (p<0.0017 for all comparisons). Additionally, the prevalence of COPD was significantly lower in both younger and older adults compared with elderly adults (p<0.017) [63]. In studies reporting in children with bronchiectasis, the prevalence of comorbid asthma ranged from 22.2 to 25.8% [65, 78] and the prevalence of sinusitis was reported to be 12.7% in a single study [79].

##### Charlson comorbidity index (CCI)

CCI scores can range from 0 to 37, with higher scores indicating a decreased estimate of 10-year survival. In this review, CCI scores ranged from 0.7 to 6.6 in studies reporting means (number of studies: 7). In one study, adults with bronchiectasis (people with CFBE excluded) who experienced ≥2 exacerbations per year were found to have significantly higher CCI scores (3.3) compared with patients who experienced less than two exacerbations per year (2.2) (p=0.001) [35]. In another study in adults with bronchiectasis (people with CFBE excluded), CCI scores increased significantly with increasing disease severity, with patients with mild (FACED score of 0–2), moderate (FACED score of 3–4) and severe (FACED score of 5–7) bronchiectasis reporting mean CCI scores of 3.9, 5.7 and 6.3, respectively [80]. No CCI scores were reported for CFBE specifically.

##### Prevalence of comorbidities in patients with bronchiectasis compared with control individuals

Several studies reported a higher prevalence of cardiovascular comorbidities. such as heart failure [81], stroke [82, 83] and hypertension [82–84] in patients with bronchiectasis compared with a matched general population or healthy controls. Conversely, several additional studies reported no significant differences [81, 85, 86]. Two large studies reported an increased prevalence of diabetes in patients with bronchiectasis compared with nonbronchiectasis control groups [83, 84]; however, three additional smaller studies reported no significant differences [81, 82, 86]. The prevalence of gastro–oesophageal reflux disease was found to be significantly higher in patients with bronchiectasis compared with matched nonbronchiectasis controls in one study [87], but no significant difference was reported in a second study [85]. Both anxiety and depression were found to be significantly more prevalent in patients with bronchiectasis compared with matched healthy controls in one study [55]. Lastly, two large studies reported an increased prevalence of asthma [84, 87] and five studies reported a significantly higher prevalence of COPD [81, 82, 84, 85, 87] in patients with bronchiectasis compared with matched nonbronchiectasis controls or the general population. A smaller study reported conflicting evidence whereby no significant difference in the prevalence of asthma in patients with bronchiectasis compared with matched controls was reported [85].

### Socioeconomic burden

#### Patient-reported outcomes

Health-related quality of life (HRQoL), fatigue, anxiety and depression were reported across several PRO measures and domains. The most frequently reported PROs are discussed in further detail in the sections below (table 2). Further details and additional PROs can be seen in the supplemental Excel file.

In a study comparing bronchiectasis (people with CFBE excluded) in different age groups (younger adults: 18–65 years; older adults: 66–75 years; elderly adults: ≥76 years), the median SGRQ total score was significantly higher in elderly adults (50.8) compared with younger adults (36.1), indicating a higher degree of limitation (p=0.017) [63].

In a study that reported Leicester Cough Questionnaire (LCQ) scores in men and women with bronchiectasis (people with CFBE excluded) separately, women had significantly lower LCQ total scores (14.9) when compared with men (17.5) (p=0.006), indicating worse quality of life [88]. Additionally, women had significantly lower scores across all three LCQ domains (p=0.014, p=0.005 and p=0.011 for physical, psychological and social domains, respectively) [88].

#### Exercise capacity

Exercise capacity in patients with bronchiectasis was reported using walking tests namely the 6-minute walk test (6MWT) and the incremental shuttle walk test (ISWT) (supplementary table S6). The 6MWT data from patients with bronchiectasis generally fell within the normal range for healthy people; however, the ISWT data was below the normal range for healthy people (supplementary table S6). Studies also reported on daily physical activity, daily sedentary time and number of steps per day in patients with bronchiectasis, and in children specifically (supplementary table S6). No data relating to disease severity were reported for CFBE specifically. Further details can be seen in the supplemental Excel file.

##### Exercise capacity in patients with bronchiectasis compared with control individuals

In one study, the ISWT distance was reported to be significantly lower in patients with NCFBE compared with healthy controls (592.6 m *versus* 882.9 m; difference of ∼290 m; p<0.001) [89]. Additionally, patients with bronchiectasis spent significantly less time on activities of moderate and vigorous intensity compared with healthy controls (p=0.030 and 0.044, respectively) [89]. Lastly, a study reported that patients with NCFBE had a significantly lower step count per day compared with healthy controls (p<0.001) [89].

#### Mortality

##### Mortality rate during study period

Mortality ranged from 0.24 to 67.6%; however, it should be noted that the study duration differed across studies. When limited to larger or multicentre studies, the mortality rate ranged from 0.24 to 28.1%. One study reported more deaths in patients with NCFBE (9.1%; 5.9-year mean follow-up period) compared with patients without bronchiectasis (0.8%; 5.4-year mean follow-up period) [84]. In one study, significantly more patients with COPD-related bronchiectasis died (37.5%) compared with other aetiologies (19.0%) (3.4-year mean follow-up period; p<0.001). After adjusting for several factors, multivariate analysis showed that the diagnosis of COPD as the primary cause of bronchiectasis increased the risk of death by 1.77 compared with the patients with other aetiologies [41]. Similarly, in another study, COPD-associated bronchiectasis was associated with higher mortality (55%) in multivariate analysis as compared with other aetiologies (rheumatic disease: 20%; post-infectious: 16%; idiopathic: 14%; ABPA: 13%; immunodeficiency: 11%) (hazard ratio 2.12, 95% CI 1.04–4.30; p=0.038; 5.2-year median follow-up period) [90].

##### Mortality rates by year

The 1-, 2-, 3-, 4- and 5-year mortality rates in patients with bronchiectasis (people with CFBE excluded, unless unspecified) ranged from 0.0 to 12.3%, 0.0 to 13.0%, 0.0 to 21.0%, 5.5 to 39.1% and 12.4 to 53.0%, respectively (number of studies: 9, 4, 7, 1 and 4, respectively). When limited to larger or multicentre studies, the 1-, 2-, 3- and 5-year mortality rates ranges were 0.4–7.9%, 3.9–13.0%, 3.7–21.0% and 12.4–53.0% (no 4-year mortality data from larger or multicentre studies). No data relating to mortality rates were reported for CFBE specifically.

Two studies reported mortality rate by bronchiectasis aetiology (people with CFBE excluded). In the first study, no significant difference in the 4-year mortality rate was reported across aetiologies (p=0.7; inflammatory bowel disease: 14.3%; post-TB: 13.4%; rheumatoid arthritis: 11.4%; idiopathic or post-infectious: 10.1%; ABPA: 6.1%; other aetiologies: 6.1%) [49]. In the second study, patients with post-TB bronchiectasis had a significantly higher 5-year mortality rate (30.0%) compared with patients with idiopathic bronchiectasis (18.0%) and other aetiologies (10.0%) (p<0.05 for both comparisons) [32].

##### In-hospital and intensive care unit mortality

In-hospital mortality ranged from 2.9 to 59.3% in patients with bronchiectasis (people with CFBE excluded, unless unspecified) hospitalised for an exacerbation or for other reasons (number of studies: 7). When limited to larger or multicentre studies, in-hospital mortality rate was reported in only one study (33.0%). One study reported mortality in bronchiectasis patients admitted to a tertiary care centre according to aetiology; in-hospital mortality was highest in patients with post-pneumonia bronchiectasis (15.8%), followed by patients with idiopathic (7.1%) and post-TB (2.6%) bronchiectasis. No deaths were reported in patients with COPD, ABPA or PCD aetiologies [42]. Intensive care unit mortality was reported in two studies and ranged from 24.6 to 36.1% [62, 91]. No data relating to mortality rates were reported for CFBE specifically.

#### Impact on family and caregivers

Only two studies discussed the impact that having a child with bronchiectasis has on parents/caregivers. In the first study, parents of children with bronchiectasis (not specified whether children with CFBE were excluded) were more anxious and more depressed according to both the Hospital Anxiety and Depression Scale (HADS) and the Centre of Epidemiological Studies depression scale, compared with parents of children without any respiratory conditions (both p<0.001; sample size of 29 participants) [53]. In the second study, parents or carers of children with bronchiectasis (multicentre study with a sample size of 141 participants; children with CFBE excluded) were asked to vote for their top five greatest concerns or worries; the most common worries or concerns that were voted for by over 15% of parents were “impact on his/her adult life in the future, long-term effects, normal life” (29.8%), “ongoing declining health” (25.5%), “the cough” (24.8%), “impact on his/her life now as a child (play, development)” (24.1%), “lack of sleep/being tired” (24.1%), “concerns over aspects of antibiotic use” (22.7%), “missing school or daycare” (17.7%) and “breathing difficulties/shortness of breath” (16.3%) [92].

#### HCRU

HCRU in terms of hospitalisations, ED visits, outpatient visits and length of stay overall and by bronchiectasis aetiology are reported in table 3. No data relating to HCRU were reported for CFBE specifically.

In a study in children with bronchiectasis (children with CFBE excluded), 30.0% of children were hospitalised at least once in the previous year [65]. The median number of hospitalisations per year was 0 (interquartile range: 0–1) [65]. In another study, the mean length of hospital stay for children with bronchiectasis was 6.7 days (standard deviation: 4.8 days) [93]. In a study comparing bronchiectasis (people with CFBE excluded) in different age groups, significantly more elderly adults (≥76 years; 26.0%) were hospitalised at least once during the first year of follow-up compared with younger adults (18–65 years; 17.0%) and older adults (66–75 years; 17.0%) (p<0.017 for both comparisons) [63]. Additionally, length of stay was found to be significantly longer in male patients (mean: 17.6 days) compared with female patients (mean: 12.5 days) (p=0.03) [94].

##### HCRU in patients with bronchiectasis compared with control individuals

Length of stay was found to be 38% higher in patients with bronchiectasis (mean: 15.4 days; people with CFBE excluded) compared with patients with any other respiratory illness (mean: 9.6 days) (p<0.001) [94]. In a study reporting on HCRU in patients with bronchiectasis (people with CFBE excluded) over a 3-year period (Germany; 2012–2015) [85], a mean of 24.7 outpatient appointments per patient were reported; there was no significant difference in the number of outpatient appointments between patients with bronchiectasis and matched controls (patients without bronchiectasis matched by age, sex and distribution, and level of comorbidities) (mean: 23.4) (p=0.12). When assessing specific outpatient appointments over the 3-year period, patients with bronchiectasis attended a mean of 9.2 general practitioner appointments, 2.9 radiology appointments, 2.5 chest physician appointments and 0.8 cardiologist appointments. Patients with bronchiectasis had significantly fewer general practitioner appointments compared with matched controls (mean: 9.8) (p=0.002); however, they had significantly more radiology appointments (mean for matched controls: 2.3) and chest physician appointments (mean for matched controls: 1.4) compared with matched controls (p<0.001 for both comparisons).

##### Hospital admission rates

In England, Wales and Northern Ireland, the crude hospital admission rate in 2013 was 88.4 (95% CI 74.0–105.6) per 100 000 person-years [91]. In New Zealand (2008–2013), the crude and adjusted hospital admission rates were 25.7 and 20.4 per 100 000 population, respectively [95]. Lastly, in Australia and New Zealand (2004–2008) the hospital admission rate ranged from 0.7 to 2.9 per person-year [96]. In all of the abovementioned studies, people with CFBE were excluded.

#### Treatment burden

In two studies, the percentage of patients with bronchiectasis receiving any respiratory medication at baseline ranged from 60.8 to 85.7% [97, 98]. Additionally, in a study comparing healthcare costs in patients with bronchiectasis before and after confirmation of *P. aeruginosa* infection, mean pharmacy visits in the year preceding diagnosis were reported to be 23.2; this increased significantly by 56.5% to 36.2 in the year post-diagnosis (p<0.0001) [99]. In another study, patients with bronchiectasis were prescribed a mean of 12 medications for bronchiectasis and other comorbidities [100]. In all of the abovementioned studies, people with CFBE were excluded. The most frequently reported respiratory treatments can be seen in supplementary table S7. These included antibiotics (including macrolides), corticosteroids, bronchodilators, mucolytics and oxygen. No treatment data were reported for CFBE specifically. Other respiratory treatments included saline, anticholinergics and leukotriene receptor antagonists (supplemental Excel file).

In studies reporting in children with bronchiectasis, 23.9% of children were receiving any bronchodilator at baseline [101], 9.0–21.7% of children were receiving inhaled corticosteroids (ICS) at baseline [101, 102], 4.3% of children were receiving oral corticosteroids at baseline [101] and 12.1% of children were receiving long-term oxygen therapy [103].

#### Medical and nonmedical indirect impacts and costs

Medical costs for bronchiectasis included overall costs, hospitalisation costs, ED visits and outpatient visit costs and costs of treatment; indirect impacts and costs included sick leave and sick pay, missed work and income loss for caregivers, and missed school or childcare for children (table 4 and the supplemental Excel file). People with CFBE were excluded from all of the studies in table 4 below. In studies reporting in currencies other than the €, costs were converted to € based on the average exchange rate for the year in which the study was conducted.

**TABLE 4:** Bronchiectasis-related medical costs and indirect impacts and costs (individual studies)

Metric	Cost	Country	Year	Comparison with matched controls	Reference
**Direct medical costs**					
Overall costs					
Mean healthcare cost per patient per year (adults)	Overall: €218 With the following comorbidities: Malignancy: €4190 Myocardial infarction: €2142 Cerebrovascular disease: €1515 TB: €1055	South Korea	2012–2017	–	[104]
Mean total direct medical expenditure per patient over a 3-year period	€18 635 (For comparison purposes, this equates to ∼€6212 per year)	Germany	2012–2015	Significantly higher total direct expenditure compared with matched controlsa (€14 237) (p<0.001)	[85]
Mean healthcare cost (maintenance treatment, exacerbations, ED visits and hospital admissions) per patient over a 1-year period (adults)	Overall: €4672 COPD-related bronchiectasis: €7449 >2 exacerbations in study year: €7521 P. *aeruginosa* infection^b^: €8654 Severe bronchiectasis^c^: €9999 ≥2 hospitalisations in study year: €16 743	Spain	2013	–	[80]
Mean cost of exacerbations (ED visits, hospital admissions and antibiotics) per patient over a 1-year period (adults)	Overall: €1491 COPD-related bronchiectasis: €3026 >2 exacerbations in study year: €3397 Severe bronchiectasis^c^: €4306	Spain	2013	–	[80]
Mean all-cause healthcare cost per patient in the year pre- and post-*P. aeruginosa* diagnosis (adults and children)	Pre-diagnosis: €27 272 Post-diagnosis: €51 033 (Significant increase in cost; p<0.0001)	USA	2007–2013	–	[99]
Hospitalisation costs: total					
Total cost of hospitalisations for adults with bronchiectasis	2004–2015: €448 948 829 2015 only: €48 606 911	Spain	2004–2015	–	[105]
Mean hospitalisation cost per patient over a 1-year period (adults)	€4666	Spain	2014–2015	–	[40]
	Overall: €1215 COPD-related bronchiectasis: €2725 >2 exacerbations in study year: €3011 Severe bronchiectasisc: €3784	Spain	2013	–	[80]
	€1874	USA	2013	–	[106]
Mean cost of hospitalisation per patient over a 3-year period	€6504 (For comparison purposes, this equates to ∼€2168 per year)	Germany	2012–2015	Significantly higher cost of hospitalisation compared with matched controls^a^ (€4184) (p<0.001)	[85]
Mean hospitalisation cost per patient in the year pre- and post-*P. aeruginosa* diagnosis (adults and children)	Pre-diagnosis: €15 379 Post-diagnosis: €27 612 (Significant increase in cost; p=0.0004)	USA	2007–2013	–	[99]
Hospitalisation costs: per hospitalisation					
Cost per hospitalisation (adults)	€2941	New Zealand	2015	–	[95]
	€1070	China	2020	–	[107]
Cost per hospitalisation (children)	€18 242	Australia	2020	–	[108]
Daily cost of hospitalisation (children)	€1508	Australia	2020	–	[108]
Hospitalisation costs: in-hospital treatments					
Mean total cost of treatments administered in the hospital ward per patient over a 1-year period (adults)	Overall: €432 Intravenous antibiotics: €234 Inhalers^d^: €59	Spain	2014–2015	–	[40]
ED visit costs: overall					
Mean ED visit cost per patient over a 1-year period (adults)	€432	Spain	2014–2015	–	[40]
	Overall: €73 COPD-related bronchiectasis: €125 >2 exacerbations in study year: €165 Severe bronchiectasis^c^: €188	Spain	2013	–	[80]
Mean ED visit cost per patient in the year pre- and post-*P. aeruginosa* diagnosis (adults and children)	Pre-diagnosis: €214 Post-diagnosis: €310 (Significant increase in cost; p<0.001)	USA	2007–2013	–	[99]
ED visit costs: treatments administered in the ED					
Mean total cost of treatments administered in the ED per patient over a 1-year period (adults)	Overall: €41 Intravenous antibiotics: €17 Inhalers: €11	Spain	2014–2015	–	[40]
Outpatient visit costs					
Mean cost of outpatient care per patient over 1-year period (adults)	€1965	USA	2013	–	[106]
Mean outpatient diagnostic and visiting costs per patient over a 3-year period	€2984 (For comparison purposes, this equates to ∼€995 per year)	Germany	2012–2015	No significant difference compared with matched controls^a^ (€2793) (p=0.27)	[80]
Mean physician office visit cost per patient in the year pre- and post-P. *aeruginosa* diagnosis (adults and children)	Pre-diagnosis: €1717 Post-diagnosis: €3076 (Significant increase in cost; p<0.001)	USA	2007–2013	–	[99]
HITH costs					
Mean HITH cost per patient over a 1-year period (adults)	€2576	Spain	2014–2015	–	[40]
Cost of prescribed treatments					
Mean cost of outpatient prescribed drugs per patient over a 3-year period	Overall: €7695 Anti-obstructive drugs: €1595 Antibiotics: €414 Mucoactive agents: €70 (For comparison purposes, this equates to ∼€2565, €532, €138 and €23 per year, respectively)	Germany	2012–2015	No significant difference in overall cost compared with matched controls^a^ (€6605) (p=0.67) Significantly higher cost of anti-obstructive drugs (p<0.001), antibiotics (p<0.001) and mucoactive agents (p=0.001) compared with matched controls^a^	[85]
Mean cost of treatments per patient over a 1-year period (adults)	Inhaled antibiotics: €2042 Inhalers (LABA, ICS, anticholinergics): €759 Oral antibiotics: €151 Home oxygen therapy: €111	Spain	2013	–	[80]
	Prescription medication (outpatient): €382	USA	2013	–	[106]
Mean cost of prescribing antibiotics per patient per year (adults)	€117	South Korea	2012–2017	–	[104]
Mean pharmacy cost per patient in the year pre- and post-*P. aeruginosa* diagnosis (adults and children)	Pre-diagnosis: €3276 Post-diagnosis: €6616 (Significant increase in cost; p<0.001)	USA	2007–2013	–	[99]
**Other medical costs**					
Mean physiotherapy cost per patient over a 1-year period (adults)	Overall: €41 >2 exacerbations in study year: €56 COPD-related bronchiectasis: €59 Severe bronchiectasis^c^: €100	Spain	2013	–	[80]
Mean cost of outpatient remedies (e.g. physiotherapy and breathing/drainage techniques) per patient over a 3-year period	Overall: €389 (For comparison purposes, this equates to ∼€130 per year)	Germany	2012–2015	Significantly higher cost of outpatient remedies compared with matched controls^a^ (€240) (p=0.02)^a^	[85]
Mean cost of outpatient medication aids (e.g. nebulisers and respiration therapy equipment) per patient over a 3-year period	Overall: €1086 (For comparison purposes, this equates to ∼€362 per year)	Germany	2012–2015	Significantly higher cost of outpatient medical aids compared with matched controls^a^ (€394) (p<0.001)^a^	[85]
Mean convalescence costs (admission to convalescence centre) per patient over a 1-year period (adults)	Overall: €52 COPD-related bronchiectasis: €126 >2 exacerbations in study year: €127 Severe bronchiectasisc: €261	Spain	2013	–	[80]
**Indirect impacts and costs**					
Sick leave, sick pay and income lost due to absenteeism					
Mean days of sick leave and mean cost of sick pay per patient over a 1-year period (adults)	Sick leave days: 13.4 Sick leave pay: €778	Spain	2014–2015	–	[40]
Mean days of sick leave and mean cost of sick pay per patient over a 3-year period	Sick leave days: 40.5 Sick leave pay^e^: €22 (For comparison purposes, this equates to ∼13.5 days and ∼€7 per year, respectively)	Germany	2012–2015	No significant difference in sick leave days or sick leave pay compared with matched controls^a^ (45.7 days; €22) (p=0.18; p=0.8)^a^	[85]
Mean cost of absenteeism per patient over a 3-year period	€4230 (For comparison purposes, this equates to ∼€1410 per year)	Germany	2012–2015	–	[85]
Missed work and lost wages for caregivers of patients with bronchiectasis					
Median number of carers who missed work (carers of children)	Primary carers: 11.6 per 100 child-months^f^ Secondary carers: 3.5 per 100 child-months^f^	Australia and New Zealand	2012–2016	–	[93]
Median number of workdays missed by carers (carers of children)	Primary carers: 3.5 days per child-year^f^ Secondary carers: 0.5 days per child-year^f^	Australia and New Zealand	2012–2016	–	[93]
Mean days of sick leave and mean cost of sick pay per carer over a 1-year period (carers of adults)	Sick leave days: 6.2 Sick leave pay: €357	Spain	2014–2015	–	[40]
Lost wages or opportunity costs for parents who stayed in hospital with their child per admission	€1614	Australia	2020	–	[108]
Missed school or childcare					
Median number of children who missed school or childcare	24.9 per 100 child-months^f^	Australia and New Zealand	2012–2016	–	[93]
Median number of days that children missed school or childcare	12 days per child-year^f^	Australia and New Zealand	2012–2016	–	[93]
Percentage of children absent from school due to respiratory symptoms in the previous year	46.9%	Australia, Alaska and New Zealand	2015–2018	–	[109]

Blank cells indicate costs for which there was no comparison with matched controls. ^a^: Matched for age, sex and comorbidities. ^b^: Defined as current or previous chronic bronchial infection with *P. aeruginosa*. ^c^: Defined as a FACED (Forced expiratory volume in 1 s, Age, Chronic Colonisation, Extension, Dyspnoea) score of 5–7. ^d^: Including bronchodilators and ICS. ^e^: Sick pay is paid out in the statutory company health insurance as a substitute wage from Day 43 of the sick leave according to Section 44 of the 5th German Social Code (this does not include the initial 6 weeks full employers pay) [85]. ^f^: Child-years and child-months take into account the number of children in the study as well as the amount of time each child was followed up for. ED: emergency department; HITH: hospital in the home; ICS: inhaled corticosteroids; LABA: long-acting beta agonists; *P*.: *Pseudomonas;* TB: tuberculosis.

## Discussion

No review to date has systematically evaluated the overall disease burden of bronchiectasis. Here, we present the first systematic literature review that comprehensively describes the clinical and socioeconomic burden of bronchiectasis overall and across individual aetiologies and associated diseases. A total of 338 publications were included in the final analysis. Together, the results indicate that the burden of clinically significant bronchiectasis on patients and their families, as well as on healthcare systems, is substantial, highlighting the urgent need for new disease-modifying therapies for bronchiectasis.

Bronchiectasis is associated with genetic, autoimmune, airway and infectious disorders. However, in many patients with bronchiectasis, an underlying aetiology cannot be identified (idiopathic bronchiectasis) [1, 3, 4]. This is supported by the results of this systematic literature review, in which up to 80.7% of patients were reported to have idiopathic bronchiectasis. The results are in line with those reported in a systematic literature review of bronchiectasis aetiology conducted by Gao *et al.* [13] (studies from Asia, Europe, North and South America, Africa and Oceania included) in which an idiopathic aetiology was reported in approximately 45% of patients with bronchiectasis, with a range of 5–82%. The maximum of 80.7% of patients with idiopathic bronchiectasis identified by this systematic literature review is much higher than in the recent report on the disease characteristics of the EMBARC where idiopathic bronchiectasis was the most common aetiology and reported in only ∼38% of patients with bronchiectasis [17]. This highlights the importance of sample size and geographic variation (80.7% reported from a single-country study with a small sample size *versus* ∼38% reported from a continent-wide study with a large sample size). Nevertheless, identifying the underlying aetiology is a recommendation of bronchiectasis guidelines as this can considerably alter the clinical management and prognosis [23, 110]. Specific therapeutic interventions may be required for specific aetiologies, such as ICS for people with asthma-related bronchiectasis, antifungal treatment for those with ABPA-associated bronchiectasis and immunoglobulin replacement therapy for those with common variable immunodeficiency-related bronchiectasis [23, 111]. Indeed, an observational study has shown that identification of the underlying aetiology affected management in 37% of people with bronchiectasis [112]. Future studies to determine the impact of identifying the underlying aetiology on management and prognosis are needed to fully understand its importance.

Patients with bronchiectasis experienced a significant symptom burden, with dyspnoea, cough, wheezing, sputum production and haemoptysis reported most commonly. These symptoms were also reported in children with bronchiectasis at slightly lower frequencies. Dealing with bronchiectasis symptoms are some of the greatest concerns from a patient's perspective. In a study assessing the aspects of bronchiectasis that patients found most difficult to deal with, sputum, dyspnoea and cough were the first, fifth and sixth most common answers, respectively [113]. Some aetiologies were reported to have a higher prevalence of certain symptoms. For example, in single studies, patients with PCD-related bronchiectasis were found to have a significantly higher prevalence of cough and wheezing [39], patients with COPD-related bronchiectasis were found to have a significantly higher prevalence of sputum production [41], and patients with post-TB bronchiectasis were found to have a higher prevalence of haemoptysis [30] compared with other aetiologies. Together, these results highlight the need for novel treatments that reduce the symptom burden of bronchiectasis. They also highlight the importance of teaching patients to perform and adhere to regular nonpharmacological interventions, such as airway clearance using physiotherapy techniques, which have been shown to improve cough-related health status and chronic sputum production [110]. Future studies assessing when airway clearance techniques should be started, and which ones are the most effective, are a research priority [113].

The burden of exacerbations in patients with bronchiectasis was high, with patients experiencing three or more exacerbations in the previous year (up to 73.6%), per year (up to 55.6%) or in the first year of follow-up (up to 32.4%). Few studies reported significant differences between aetiologies. Importantly, exacerbations are the second-most concerning aspect of bronchiectasis from the patient's perspective [113]. Patients with frequent exacerbations have more frequent hospitalisations and increased 5-year mortality [114] and exacerbations are also associated with poorer quality of life [114, 115]. Therefore, prevention of exacerbations is of great importance in the management of bronchiectasis [116]. The exact cause of exacerbations in bronchiectasis (believed to be multifactorial) is not fully understood due a lack of mechanistic studies [116]. Future studies into the causes and risk factors for exacerbations [113] may lead to improvements in their prevention.

Many patients with bronchiectasis, including children, experienced chronic infections with bacterial pathogens such as *P. aeruginosa*, *H. influenzae*, *Sta. aureus* and *Str. pneumoniae* as well as non-tuberculous mycobacteria. Importantly, *P. aeruginosa* infection was significantly associated with more severe disease, reduced lung function and quality of life, and increased exacerbations, hospital admission, morality, HCRU and healthcare costs. Due to the clear and consistent association between *P. aeruginosa* and poor outcomes, patients with chronic *P. aeruginosa* colonisation should be considered to be at a higher risk of bronchiectasis-related complications [110]. Additionally, regular sputum microbiology screening should be performed in people with clinically significant bronchiectasis to detect new isolation of *P. aeruginosa* [110]; in which case, patients should be offered eradication antibiotic treatment [23]. Eradication of *P. aeruginosa* is not only of clinical importance, but also of economic importance due to the associated HCRU and healthcare costs. As such, a better understanding of the key factors leading to *P. aeruginosa* infection is a priority for future research [113].

Bronchiectasis markedly impacted HRQoL across several PROs including the SGRQ, Quality of Life–Bronchiectasis score, LCQ, COPD Assessment Test and Bronchiectasis Health Questionnaire. In children with bronchiectasis, significantly lower quality of life (according to the Paediatric Quality of Life Inventory score) compared with age-matched controls was reported [53]. The majority of studies reporting HRQoL in individual aetiologies and associated diseases either reported in a single aetiology, did not perform any statistical analyses to compare aetiologies, or reported no significant differences across aetiologies. Patients also experienced mild-to-moderate anxiety and depression according to the HADS-Anxiety, HADS-Depression and 9-question Patient Health Questionnaire scores, with very limited data reported in individual aetiologies. When compared with healthy controls, anxiety and depression were found to be significantly more prevalent in patients with bronchiectasis [55]. Additionally, exercise capacity was reduced, with patients with bronchiectasis reported to spend significantly less time on activities of moderate and vigorous intensity and have a significantly lower step count per day compared with healthy controls [89]. Improvements in anxiety, depression and exercise capacity are important priorities for people with bronchiectasis; in a study assessing the aspects of bronchiectasis that patients found most difficult to manage, “not feeling fit for daily activities”, anxiety and depression were the fourth, eighth and ninth most common answers, respectively [113].

The studies relating to HCRU and costs in this review were heterogeneous in terms of methodology, time period, country and currency, making them challenging to compare. Nevertheless, this study found that HCRU was substantial, with patients reporting a maximum of 1.3 hospitalisation, 1.3 ED and 21.0 outpatient visits per year. Length of stay was found to be significantly longer in patients with bronchiectasis compared with patients with any other respiratory illness in one study [91]. In another study, patients with bronchiectasis reported significantly more specialist appointments (radiologist appointments and chest physician appointments) compared with matched controls [85]. Patients with bronchiectasis also experienced a significant treatment burden, with up to 36.4, 58.0 and 83.0% of patients receiving long-term inhaled antibiotics, oral antibiotics and macrolides, respectively, up to 80.4% receiving long-term ICS and up to 61.7% and 81.4% receiving long-term long-acting muscarinic antagonists and long-acting beta agonists, respectively. Wide ranges of treatment use were reported in this study, which may reflect geographic variation in treatment patterns. Heterogeneous treatment patterns across Europe were observed in the EMBARC registry data with generally higher medication use in the UK and Northern/Western Europe and lower medication use in Eastern Europe (inhaled antibiotics: 1.8–8.9%; macrolides: 0.9–24.4%; ICS: 37.2–58.5%; long-acting beta agonists: 42.7–52.8%; long-acting muscarinic antagonists: 26.5–29.8%) [17]. Similarly, data from the Indian bronchiectasis registry indicate that the treatment of bronchiectasis in India is also diverse [19]. Furthermore, in a comparison of the European and Indian registry data, both long-term oral and inhaled antibiotics were more commonly used in Europe compared with India [19].

Cost varied widely across studies. However, patients, payers and healthcare systems generally accrued substantial medical costs due to hospitalisations, ED visits, outpatient visits, hospital-in-the-home and treatment-related costs. Other medical costs incurred included physiotherapy and outpatient remedies (including breathing or drainage techniques), outpatient medical aids (including nebulisers and respiration therapy equipment) and the cost of attending convalescence centres. Only one study compared the medical costs in patients with bronchiectasis and matched controls (age, sex and comorbidities) and found that patients with bronchiectasis had significantly higher total direct medical expenditure, hospitalisation costs, treatment costs for certain medications and costs associated with outpatient remedies and medical aids [85]. Bronchiectasis was also associated with indirect impacts and costs, including sick leave, sick pay and income lost due to absenteeism and missed work, and lost wages for caregivers of patients with bronchiectasis. Children with bronchiectasis also reported absenteeism from school or childcare.

Our findings regarding HRCU and costs in bronchiectasis are mirrored by a recent systematic literature review by Roberts *et al*. [117] estimating the annual economic burden of bronchiectasis in adults and children over the 2001–2022 time period. Roberts *et al*. [117] found that annual total healthcare costs per adult patient ranged from €3027 to €69 817 (costs were converted from USD to € based on the average exchange rate in 2021), predominantly driven by hospitalisation costs. Likewise, we report annual costs per patient ranging from €218 to €51 033, with annual hospital costs ranging from €1215 to €27 612 (adults and children included) (table 4). Further, Roberts *et al*. [117] reports a mean annual hospitalisation rate ranging from 0.11 to 2.9, which is similar to our finding of 0.03–1.3 hospitalisations per year (table 3). With regard to outpatient visits, Roberts *et al*. [117] reports a mean annual outpatient respiratory physician attendance ranging from 0.83 to 6.8 visits, whereas we report a maximum of 21 visits per year (table 3). It should be noted, however, that our value is not restricted to visits to a respiratory physician. With regard to indirect annual costs per adult patient, Roberts *et al*. [117] reports a loss of income because of illness of €1109–€2451 (costs were converted from USD to € based on the average exchange rate in 2021), whereas we report a figure of ∼€1410 (table 4). Finally, burden on children is similarly reported by us and Roberts *et al*. [117], with children missing 12 days of school per year per child (table 4).

### Limitations of this review and the existing literature

Due to the nature of this systematic literature review, no formal statistical analyses or formal risk of bias assessments were performed.

Several limitations within the existing literature were identified. Firstly, the vast majority of studies reported patients with NCFBE overall, with limited availability of literature reporting on individual aetiologies and associated disease. Furthermore, where this literature was available, it was limited to a handful of individual aetiologies and associated diseases, and in many of these studies, no statistical analyses to compare different aetiologies and associated disease were performed. Additionally, the methods used to determine aetiologies within individual studies may have differed. Literature on NCFBE and CFBE has traditionally been very distinct; as such, most of the studies included in this review have excluded people with CF. As the general term “CF lung disease” was not included in our search string in order to limit the number of hits, limited data on CFBE are included in this review. Bronchiectasis remains largely under-recognised and underdiagnosed, thus limiting the availability of literature. There is a particular knowledge gap with respect to paediatric NCFBE; however, initiatives such as the Children's Bronchiectasis Education Advocacy and Research Network (Child-BEAR-Net) (www.improvebe.org) are aiming to create multinational registries for paediatric bronchiectasis.

There were variations in the amount of literature available for the individual burdens. While there was more literature available on the clinical burden of bronchiectasis, economic data (related to both medical costs and indirect costs) and data on the impact of bronchiectasis on families and caregivers, were limited. Additionally, cost comparisons across studies and populations were difficult due to differences in cost definitions, currencies and healthcare systems.

Sample sizes of the studies included in this systematic literature review varied greatly, with the majority of studies reporting on a small number of participants. Furthermore, many of the studies were single-centre studies, thus limiting the ability to make generalisations about the larger bronchiectasis population, and cross-sectional, thus limiting the ability to assess the clinical and socioeconomic burden of bronchiectasis over a patient's lifetime. Furthermore, there may be potential sex/gender bias in reporting that has not been considered in this systematic literature review.

Finally, for many of the reported outcomes, data varied greatly across studies, with wide estimates for the frequency of different aetiologies and comorbidities as well as disease characteristics such as exacerbations and healthcare costs noted. This reflects the heterogeneity of both the study designs (including sample size and inclusion and exclusion criteria) and the study populations themselves. Additionally, the use of non-standardised terms across articles posed a limitation for data synthesis. Systematic collection of standardised data across multiple centres, with standardised inclusion and exclusion criteria such as that being applied in international registries, is likely to provide more accurate estimates than those derived from small single-centre studies.

## Conclusions

Collectively, the evidence identified and presented in this systematic literature review show that bronchiectasis imposes a significant clinical and socioeconomic burden on patients and their families and employers, as well as on healthcare systems. Disease-modifying therapies that reduce symptoms, improve quality of life, and reduce both HCRU and overall costs are urgently needed. Further systematic analyses of the disease burden of specific bronchiectasis aetiologies and associated disease (particularly PCD-, COPD- and post-TB-associated bronchiectasis, which appear to impose a greater burden in some aspects) and paediatric bronchiectasis (the majority of data included in this study were obtained from adults) may provide more insight into the unmet therapeutic needs for these specific patient populations.

**Questions for future research**

Further research into the clinical and socioeconomic burden of bronchiectasis for individual aetiologies and associated diseases is required.

## Supplementary material

10.1183/16000617.0049-2024.Supp1 **Please note:** supplementary material is not edited by the Editorial Office, and is uploaded as it has been supplied by the author.
Supplementary figures and tables ERR-0049-2024.SUPPLEMENT
Supplementary Excel file ERR-0049-2024.SUPPLEMENT
