# Supplementary material for: A systematic literature review of the clinical and socioeconomic burden of bronchiectasis
Source: Eur Respir Rev. 2024 Sep 4;33(173):240049. doi: 10.1183/16000617.0049-2024 (PMC11372470; doi:10.1183/16000617.0049-2024)
Supplement: Supplementary file 1 [file ERR-0049-2024.SUPPLEMENT.pdf]

SUPPLEMENTARY MATERIAL

A systematic literature review of the clinical and socioeconomic burden in patients with bronchiectasis

Supplementary Table S1. Literature review search terms

Topic	Search	Search string
**Bronchiectasis disease search**	S1	"bronchiectasis" OR ("asthma" AND "bronchiectasis") OR ("COPD" AND "bronchiectasis") OR (“chronic obstructive pulmonary disease” AND “bronchiectasis”) OR (“CF” AND “bronchiectasis”) OR (“cystic fibrosis” AND “bronchiectasis”) OR (“AATD” AND “bronchiectasis”) OR (“A1AD” AND “bronchiectasis”) OR (“alpha-1 antitrypsin deficiency” AND “bronchiectasis”) OR (“α1-antitrypsin deficiency” AND “bronchiectasis”) OR (“PCD” AND “bronchiectasis”) OR (“primary ciliary dyskinesia” AND “bronchiectasis”) OR (“common variable immunodeficiency” AND “bronchiectasis”) OR (“CVID” AND “bronchiectasis”) OR (“rheumatoid arthritis” AND “bronchiectasis”) OR (“RA” AND “bronchiectasis”) OR (“inflammatory bowel disease” AND “bronchiectasis”) OR (“IBD” AND “bronchiectasis”) OR (“systemic sclerosis” AND “bronchiectasis”) OR (“SSc” AND “bronchiectasis”) OR (“infectious disease” AND “bronchiectasis”) OR (“post-infection” AND “bronchiectasis”) OR (“tuberculosis” AND “bronchiectasis”) OR (“TB” AND “bronchiectasis”) OR (“NTM” AND “bronchiectasis”) OR (“nontuberculous mycobacterial” AND “bronchiectasis”) OR (“nontuberculous mycobacteria” AND “bronchiectasis”) OR (“pneumonia” AND “bronchiectasis”) OR (“aspiration” AND “bronchiectasis”) OR (“human immunodeficiency virus” AND “bronchiectasis”) OR (“HIV” AND “bronchiectasis”) OR (“allergic bronchopulmonary aspergillosis” AND “bronchiectasis”) OR (“ABPA” AND “bronchiectasis”) OR (“immunodeficiency” AND “bronchiectasis”) OR (“idiopathic” AND “bronchiectasis”) OR (“CTD” AND “bronchiectasis”) OR (“connective tissue disease” AND “bronchiectasis”) OR (“collagen disease” AND “bronchiectasis”) OR (“sinonasal disease” AND “bronchiectasis”) OR (“rhinosinusitis” AND “bronchiectasis”
**Clinical burden search**	S2	“morbidity” OR “symptom burden” OR “burden of symptoms” OR “clinical burden” OR “treatment burden” OR “disease burden” OR “sequelae” OR “sequela” OR “consequence” OR “consequences” OR “complication” OR “complications” OR “dysfunction” OR “dysfunctions” OR “dysfunctional” OR “impairment” OR “impairments” OR “deficit” OR “deficits” OR “disability” OR “disabilities” OR “pulmonary exacerbation” OR “pulmonary exacerbations”
**Humanistic burden search**	S3	“International Classification of Functioning, Disability and Health” OR “quality of life” OR “value of life” OR “short form 36” OR “patient-reported outcome” OR “patient reported outcomes” OR “patient preference” OR “patient preferences” OR “questionnaire” OR “questionnaires” OR “quality adjusted life years” OR “health status” OR “health status indicator” OR “health status indicators” OR “caregiver burden” OR “societal burden” OR “family burden” OR “patient burden” OR “unmet need” OR “disability adjusted” OR “disability-adjusted” OR “dartmouth coop” OR “Duke health profile” OR “EQ 5D” OR “ED-5D” OR “EQ” OR “EuroQoL” OR “daily activity” OR “functional status” OR “reduced function” OR “impaired function” OR “decreased function” OR “health-related quality of life” OR “HRQOL” OR “Nottingham health” OR “NHP” OR “PQOL” OR “perceived quality

		of life” OR “QLS” OR “quality of life scale” OR “wellbeing” OR “well-being” OR “QWB” OR “quality of well-being” OR “short form” OR “short-form” OR “shortform” OR “SF6” OR “SF8” OR “SF12” OR “SF20” OR “SF36” OR “sickness impact” OR “SIP” OR “patient reported” OR “patient-reported” OR “self reported” OR “self-reported” OR “quality adjusted” OR “QALY” OR “quality-adjusted” OR “life year” OR “life-year” OR “health year” OR “health-year” OR “willingness to pay” OR “willingness-to-pay” OR “WTP” OR “healthy utility index” OR “hui” OR “hui1” OR “hui2” OR “hui3” OR “standard gamble” OR “time trade off” OR “TTO” OR “health assessment questionnaire” OR “health assessment questionnaires” OR “HAQ” OR “Quality of Life Questionnaire-Bronchiectasis” OR “QOL-B” OR “St. George's Respiratory Questionnaire” OR “SGRQ” OR “Leicester Cough Questionnaire” OR “LCQ” OR “Cough and Sputum Assessment Questionnaire” OR “QASA-Q” OR “Visual Analogue Scales” OR “VAS”
**Economic burden search**	S4	“cost of Illness” OR “fees and charges” OR “employer health costs” OR “efficiency” OR “presenteeism” OR “absenteeism” OR “health expenditures” OR “salary” OR “salaries” OR “fringe benefits” OR (cost* NEAR/3 (“medical” OR “direct” OR “indirect” OR “drug” OR “drugs” OR “treatment” OR “treatments” OR “medication” OR “medications” OR “pharmaceutical” OR “hospital” OR “emergency” OR “outpatient” OR “inpatient” OR “ambulatory” OR “primary care” OR “practitioner” OR “device” OR “informal” OR “economic” OR “societal” OR “intangible” OR “caregiver” OR “physician” OR “specialist” OR “healthcare” OR “health care” OR “annual” OR “clinic”)) OR ((“burden” OR “impact”) NEAR/3 (“cost” OR “costs” OR “economic” OR “caregiver” OR “caregivers” OR “family” OR “families” OR “society” OR “societal” OR “employee” OR “employer”)) OR “productivity” OR “medical leave” OR “sick day” OR “sick leave” OR “sickness absence” OR “work absence” OR “work incapacity” OR “work leave” OR “disability absence” OR “resource utilization” OR “resource utilisation” OR “health care utilization” OR “healthcare utilization” OR “health care utilisation” OR “healthcare utilisation” OR “hospitalization” OR “hospitalisation” OR “length of stay” OR “LOS”
**Clinical burden in bronchiectasis**		S1 AND S2
**Humanistic burden in bronchiectasis**		S1 AND S3
**Economic burden in bronchiectasis**		S1 AND S4

Supplementary Table S2. Disease severity of bronchiectasis overall (including the proportions of patients with mild, moderate and severe disease) and in individual bronchiectasis aetiologies as measured by BSI, FACED and E-FACED scores

Disease severity index	Score components	Range of scores (mean or median)	Score by aetiology	Proportion of patients (range of %)			Number of studies
				Mild	Moderate	Severe	
**BSI**	Radiological severity, FEV_1, MRC dyspnoea score, bacterial colonisation (*Pseudomonas aeruginosa* or other pathogenic bacteria), prior hospital admission and exacerbations. A BSI score of 0–4 indicates mild bronchiectasis, 5–8 indicates moderate bronchiectasis and >9 indicates severe bronchiectasis	4.8–15.4[a]<br>2.0–14.0[b]	Post-TB (10.5)[c], idiopathic (8.1), other aetiologies (7.0)<br>Rheumatic disease (9.8), immunodeficiency (8.6), post-TB (8.3), ABPA (7.6), post-pneumonia (6.7)[d]<br>COPD (9.0), post-infectious/idiopathic (8.0–8.4), other aetiologies (8.0), AATD (7.8), post-TB (7.0), post-infectious (7.0), asthma (6.0), idiopathic (6.0), immune deficiency (6.0), ABPA (5.0)[e]	3.8–80.5<br>[3.8–49.5]	13.8–48.1<br>[21.0–48.1]	2.4–78.7<br>[23.3–78.7]	77
**FACED**	FEV_1, age, chronic colonisation (with *P. aeruginosa*), extension (number of lobes affected) and dyspnoea.<br>A FACED score of 0–2, 3–4 and 5–7 indicates mild, moderate and severe disease, respectively	1.1–5.6[b]<br>1.0–4.0[c]	Post-TB (3.8)[c], idiopathic (2.7), other aetiologies (2.4)	5.9–77.8<br>[25.5–75.3]	22.2–78.4<br>[24.7–55.0]	0.0–45.4<br>[0.0–41.8]	29
**E-FACED**	FEV_1, age, chronic colonisation (with *P. aeruginosa*), extension (number of lobes affected), dyspnoea and exacerbations. Mild, moderate and severe disease are indicated by E-FACED scores of 0–3, 4–6 and 7–9, respectively	2.3–5.0[b]<br>2.5–5.0[c]	Post-TB (4.8)[c], idiopathic (3.1), other aetiologies (2.7)	14.9–73.9<br>[14.9–71.9]	21.7–44.7<br>[26.0–44.7]	0.9–40.4<br>[2.1–40.4]	11

Square brackets indicate data from larger or multicentre studies only. All data from individual studies are available in the supplementary Excel file.

[a]Studies reporting mean.

[b]Studies reporting median.

[c]BSI, FACED and E-FACED scores were significantly higher in patients with post-TB bronchiectasis compared with idiopathic bronchiectasis and other aetiologies ($p<0.05$ for all comparisons) [1].

[d]No significant difference in BSI score between aetiologies (p=0.798) [2]. Rheumatic disease includes RA, systemic lupus erythematosus, primary Sjögren's syndrome, vasculitis and ankylosing spondylitis.

[e]No statistical analyses performed [3-6]. 'Other' includes RA, PCD, GERD and NTM infection.

AATD, alpha-1 antitrypsin deficiency; ABPA, allergic bronchopulmonary aspergillosis; BSI, Bronchiectasis Severity Index; COPD, chronic obstructive pulmonary disease; E-FACED, Exacerbations, FEV_1, Age, Chronic Colonization, Extension, Dyspnoea; FACED, FEV_1, Age, Chronic Colonization, Extension, Dyspnoea; FEV_1, forced expiratory volume in 1 second; GERD, gastro-oesophageal reflux disease; MRC, Medical Research Council; NTM, non-tuberculous mycobacteria; PCD, primary ciliary dyskinesia; RA, rheumatoid arthritis; TB, tuberculosis.

Supplementary Table S3. Lung function in patients with bronchiectasis overall, in children with bronchiectasis and in individual bronchiectasis aetiologies

<table>
<tr><th></th><th>Lung function overall</th><th>Lung function in children</th><th>Lung function by aetiology</th><th>Number of studies</th></tr>
<tr><td rowspan="16">ppFEV_1 (%)</td><td rowspan="16">37.0–96.1[a]
[40.1–92.0][a]
30.0–96.0[b]
[30.0–90.0][b]</td><td rowspan="16">63.0–91.5[a]
85.0–91.3[b]</td><td>Post-TB (62.0), idiopathic (67.0), other aetiologies (69.0)[c]</td><td rowspan="16">189</td></tr>
<tr><td>Post-TB (63.0), other aetiologies (78.0)[d]</td></tr>
<tr><td>Idiopathic or post-infectious (54.4–78.7)[e]</td></tr>
<tr><td>CF (60.8)[f]</td></tr>
<tr><td>PCD (70.0; 81.0), other aetiologies (66.0; 83.2)[g]</td></tr>
<tr><td>AATD (70.2)[h]</td></tr>
<tr><td>COPD (45.7), other aetiologies (69.4)[i]</td></tr>
<tr><td>COPD (51.0–61.0)[j]</td></tr>
<tr><td>COPD (51.0), idiopathic (76.0), RA (76.0)[k]</td></tr>
<tr><td>HIV (53.5–56.0)[l]</td></tr>
<tr><td>COPD (46.8), asthma (63.4), post-infectious (65.6), PCD (67.1), idiopathic (67.3), systemic diseases (71.4), PID (77.7)[m]</td></tr>
<tr><td>ABPA (56.7), idiopathic (58.4), post-TB (58.7), rheumatic disease (59.9), post-pneumonia (62.7), immunodeficiency (63.1)[n]</td></tr>
<tr><td>ILD (42.7), recurrent aspiration/GERD (45.5), PID (54.7), SID (56.6), post-infectious bronchiolitis obliterans (62.1), PCD (64.1), CHD (68.1), post-TB (69.9), post-infectious (71.8)[o]</td></tr>
<tr><td>PCD (57.2), idiopathic (70.5), AATD (74.8), CVID (75.0)[p]</td></tr>
<tr><td>COPD (42.0), genetic/congenital (45.0), autoimmune (52.0), post-infectious (55.0), immunodeficiency (72.0), idiopathic (74.0), asthma (84.0)[q]</td></tr>
<tr><td>COPD (47.0), other aetiologies (51.9), idiopathic (56.2), post-infectious (57.2), PCD (60.1)[r]</td></tr>
<tr><td></td><td></td><td></td><td>COPD (47.8), post-infectious (52.8), ABPA (55.4), idiopathic (58.7), other aetiologies (60.6), asthma (63.2), post-TB (63.9)[s]</td><td></td></tr>
<tr><td rowspan="2">FEV_1 (L)</td><td rowspan="2">1.3–2.3[a]
[1.5–2.3][a]
1.0–1.9[b]
[1.0–1.9][b]</td><td rowspan="2">–</td><td>Idiopathic or post-infectious (1.4–2.1)[e]</td><td rowspan="2">56</td></tr>
<tr><td>CF (2.1)[f]</td></tr>
<tr><td rowspan="3">ppFVC</td><td rowspan="3">53.8–105.7[a]
[57.3–98.5][a]
41.0–88.0[b]</td><td rowspan="3">63.3–87.4[a]
77.0–98.0[b]</td><td>Post-TB (68.0), other aetiologies (80.0)[d]</td><td rowspan="3">107</td></tr>
<tr><td>PCD (85.0; 90.1), other aetiologies (80.0; 88.2)[g]</td></tr>
<tr><td>COPD (60.2), other aetiologies (72.8)[i]</td></tr>
</table>

	[41.0–85.6][b]		HIV (45.0–49.0)[l]	
			Rheumatic disease (72.0), post-TB (72.2), idiopathic (74.2), ABPA (74.3), immunodeficiency (75.9), post-pneumonia (76.7)[n]	
			ILD (37.2), recurrent aspiration/GERD (40.5), SID (57.8), PID (61.7), PCD (64.8), post-infectious bronchiolitis obliterans (72.6), post-infectious (72.9), CHD (73.2), post-TB (74.0)[o]	
			PCD (75.1), idiopathic (76.8), CVID (84.0), AATD (85.3)[p]	
			COPD (57.9), idiopathic (63.9), post-infectious (64.6), other aetiologies (66.6), PCD (78.3)[r]	
**FVC (L)**	1.5–3.2[a]<br>[2.3–3.1][a]<br>2.5–2.8[b]	–	Idiopathic or post-infectious (2.3–3.4)[e]	35
			CF (3.2)[f]	
**FEV_1/FVC**	49.4–86.9[a]<br>[53.1–82.0][a]<br>53.7–88.3[b]<br>[60.0–88.3][b]	78.2–81.0[a]<br>93.0[b]	Post-TB (72.0), other aetiologies (72.0)[d]	78
			PCD (79.0), other aetiologies (84.0)[t]	
			Idiopathic or post-infectious (59.7–67.4)[e]	
			COPD (55.3), other aetiologies (71.0)[i]	
			PCD (63.0), CVID (67.8), AATD (68.4), idiopathic (70.3)[p]	
			Post-TB (74.0), PID (82.5), post-infectious (86.5), post-infectious bronchiolitis obliterans (86.6), PCD (88.2), CHD (92.7), SID (98.0), recurrent aspiration/GERD (113.3), ILD (115.0)[o]	
			COPD (51.0–68.0)[j]	
			Idiopathic (62.5), ABPA (62.7), post-pneumonia (64.1), post-TB (64.2), immunodeficiency (65.4), rheumatic disease (66.2)[n]	
			COPD (56.5), asthma (66.4), post-infectious (69.6), systemic diseases (70.8), idiopathic (72.8), PCD (73.2), PID (77.9)[m]	
**LCI**	9.0–12.8[a]	–	–	2

Square brackets indicate data from larger or multicentre studies only. Blank cells indicate lung function indices for which data were not reported in children or in individual aetiologies. All data from individual studies are available in the supplementary Excel file.

[a]Studies reporting mean. Normal range of LCI reported in these studies: 6–7 or less. Inert gas used was sulphur hexafluoride.

[b]Studies reporting median.

[c]Patients with post-TB bronchiectasis had a significantly lower $ppFEV_1$ compared with patients with other aetiologies ($p<0.05$); however, there was no significant difference when compared with idiopathic bronchiectasis (no p-value given) [1].

[d]Patients (admitted to hospital for an exacerbation) with post-TB bronchiectasis had significantly lower ppFEV$_1$ compared with patients with other aetiologies (p=0.005); however, no significant difference in ppFVC or FEV$_1$/FVC was reported (p=0.05 and p=0.42, respectively) [7].

[e]Studies reporting in patients with idiopathic or post-infectious bronchiectasis; no comparison with other aetiologies [8-16].

[f]Study reported in patients with CF-related bronchiectasis; no comparison with other aetiologies [17].

[g]In one study, ppFEV$_1$ was lower in patients with PCD-related bronchiectasis compared with other aetiologies [18], while in a second study the opposite was true [19]; ppFVC was higher in patients with PCD-related bronchiectasis compared with other aetiologies in both studies [18, 19]. However, no statistical analyses were carried out in either study.

[h]Study reported in patients with AATD-related bronchiectasis; no comparison with other aetiologies [20].

[i]Patients with COPD-related bronchiectasis had significantly lower ppFEV$_1$, ppFVC and FEV$_1$/FVC compared with other aetiologies (p<0.001 for all comparisons) [21].

[j]Study reported in patients with COPD-related bronchiectasis of different severities; no comparison with other aetiologies [22].

[k]Patients with COPD-related bronchiectasis had significantly lower ppFEV$_1$ compared with idiopathic and RA-associated bronchiectasis (p<0.05 for both comparisons) [23].

[l]Study reporting in children with HIV-related bronchiectasis; no comparison with other aetiologies [24].

[m]Significant difference between aetiologies reported (p<0.001; comparison of all aetiologies, no head-to-head comparisons performed) [25].

[n]No significant differences between aetiologies for ppFEV$_1$ (p=0.963), ppFVC (p=0.920) or FEV$_1$/FVC (p=0.889) [2]. Rheumatic disease includes RA, systemic lupus erythematosus, primary Sjögren’s syndrome, vasculitis and ankylosing spondylitis.

[o]Study reported in paediatric patients with bronchiectasis (mean age: 9.2 years; age range: 0–24 years); no statistical analyses performed.

[p]Patients with PCD-related bronchiectasis had a significantly lower ppFEV$_1$ compared with patients with AATD-related, CVID-related and idiopathic bronchiectasis (p<0.0001); however, ppFVC was significantly lower in patients with PCD-related and idiopathic bronchiectasis compared with AATD-related bronchiectasis (p=0.007). FEV$_1$/FVC was significantly lower in patients with PCD-related bronchiectasis compared with idiopathic bronchiectasis and AATD-related bronchiectasis (p<0.001) [5].

[q]Significant differences between patients with COPD-related, genetic or congenital versus idiopathic bronchiectasis (p=0.0412) [26].

[r]No significant difference between aetiologies (no p-value given) [27].

[s]No statistical analyses performed [6]. ‘Other’ includes RA, PCD, GERD and NTM infection.

[t]FEV$_1$/FVC was lower in patients with PCD-related bronchiectasis compared with other aetiologies; however, no statistical analyses were carried out [18].

AATD, alpha-1 antitrypsin deficiency; ABPA, allergic bronchopulmonary aspergillosis; CF; cystic fibrosis; CHD, congenital heart disease; COPD, chronic obstructive pulmonary disease; CVID, common variable immunodeficiency; FEV$_1$, forced expiratory volume in 1 second; FVC, forced vital capacity GERD, gastro-oesophageal reflux disease; HIV, human

immunodeficiency virus; ILD, interstitial lung disease; LCI, lung clearance index; NTM, non-tuberculous mycobacteria; PCD, primary ciliary dyskinesia; PID, primary immunodeficiency; ppFEV$_1$, per cent predicted forced expiratory volume in 1 second; ppFVC, per cent predicted forced vital capacity; RA, rheumatoid arthritis; SID, secondary immunodeficiency; TB, tuberculosis.

Supplementary Table S4. Most commonly reported pathogens isolated from patients with bronchiectasis overall, in children with bronchiectasis and in individual bronchiectasis aetiologies

Chronic infection or acute infection	Prevalence overall (range of %)	Prevalence in children (range of %)	Prevalence by aetiology (% or range of %)	Number of studies
**Bacterial infections**				
P. aeruginosa	0.0–96.6[a] [0.8–85.3]	3.7–22.2	Idiopathic or post-infectious (25.0–54.7)[b]	132
			CF (81.3), NCFBE (14.7)[c]	
			CF (27.3–74.2)[d]	
			Post-TB (25.6), idiopathic (42.1), other aetiologies (22.9)[e]	
			Post-TB (4.3), other aetiologies (2.0)[f]	
			PCD (4.2; 38.0), other aetiologies (11.8; 20.0)[g]	
			PCD (63.5)[h], AATD (33.3), idiopathic (28.8) and CVID (25.0)	
			COPD (24.7)[i], other aetiologies (19.8)	
			COPD (27.0–45.0)[j]	
			COPD (24.1), idiopathic (14.7), RA (14.3)[k]	
			AATD (23.4)[l]	
			IBD (18.8), other aetiologies (22.8)[m]	
			PCD (45.0), systemic diseases (27.6), COPD (24.4), post-infectious (23.0), idiopathic (17.2), asthma (12.7), PID (11.8)[n]	
			Post-infectious (other than TB; 6.8), COPD (6.0), asthma (5.9), idiopathic (4.3), GERD (4.1), CTD (3.9), immunodeficiency (3.8), post-TB (3.8)[o]	
			Tumour (100.0), COPD (65.0), genetic/congenital (57.0), asthma (50.0), autoimmune (50.0), post-infectious (40.0), immunodeficiency (31.0), idiopathic (30.0)[p]	
			ABPA (9.7–42.9), PCD (42.9), COPD (16.4–38.9), post-TB (13.5–36.8), post-pneumonia (31.6), idiopathic (14.1–28.6), other aetiologies (22.5), post-infectious (13.6), asthma (9.3)[q]	
H. influenzae	0.0–57.4 [0.8–34.6]	29.9–100.0	Idiopathic or post-infectious (23.3–25.0)[b]	70
			PCD (8.7; 29.2)[r], other aetiologies (4.0; 17.6)	
			Post-TB (2.2), other aetiologies (2.0)[f]	
			COPD (8.2)[i], other aetiologies (12.2)	
			COPD (22.0–35.0)[j]	

			CTD (5.0), asthma (4.8), post-infectious (other than TB; 4.4), COPD (3.3), GERD (3.1), immunodeficiency (2.9), idiopathic (2.7), post-TB (1.9)[o]	
			PCD (23.3), PID (19.1), asthma (12.7), idiopathic (10.6), post-infectious (10.2), COPD (8.1), systemic diseases (3.4)[n]	
			Genetic/congenital (50.0), asthma (1.9–25.0), idiopathic (1.3–26.0), post-infectious (0.6–29.0), post-TB (0.1–5.3), ABPA (0.0), COPD (0.0–35.0), other aetiologies (0.0), immunodeficiency (46.0), autoimmune (33.0), tumour (33.0), post-pneumonia (10.5), ABPA (7.1), PCD (0.0)[s]	
H. parainfluenzae	0.0–11.3<br>[1.3]	5.1	CTD (2.8), asthma (2.3), idiopathic (1.5), post-infectious (other than TB; 1.4), immunodeficiency (1.0), GERD (1.0), post-TB (0.9), COPD (0.8)[o]	7
S. aureus[t]	0.0–49.5<br>[0.6–23.3]	0.5–11.5	PCD (4.2; 8.7)[r], other aetiologies (5.9; 9.3)	53
			COPD (0.6)[i], other aetiologies (2.3)	
			COPD (11.0–30.0)[j]	
			AATD (9.1)[l]	
			CTD (1.7), post-infectious (other than TB; 1.4), immunodeficiency (1.0), post-TB (0.8), COPD (0.7), idiopathic (0.6), GERD (0.5), asthma (0.4)[o]	
			AATD (19.4), PCD (16.9), idiopathic (9.8), CVID (0.0)[h]	
			Autoimmune (50.0), genetic/congenital (36.0), idiopathic (1.1–35.0), COPD (0.9–30.0), post-infectious (2.0–16.0), immunodeficiency (15.0), post-TB (4.4), asthma (0.0), tumour (0.0), ABPA (0.0), other aetiologies (0.0)[s]	
S. pneumoniae	0.0–32.9<br>[0.0–6.7]	1.6–47.1	Idiopathic or post-infectious (3.1–11.7)[b]	39
			PCD (4.0; 12.5)[r], other aetiologies (0.0 in both)	
			COPD (3.8)[i], other aetiologies (5.4)	
			COPD (8.0–16.0)[j]	
M. catarrhalis	0.0–18.5<br>[0.0–5.9]	0.0–14.4	Idiopathic or post-infectious (4.8–8.3)[b]	35
			COPD (0.6)[i], other aetiologies (1.3)	
			COPD (0.0–14.0)[j]	
			Immunodeficiency (15.4), genetic/congenital (14.3), COPD (0.0–13.0), idiopathic (1.3–4.3), post-infectious (1.6–2.0), post-TB (0.9), ABPA (0.5), asthma (0.0), autoimmune (0.0), tumour (0.0), other aetiologies (0.0)[s]	

**Mycobacterial infections**				
Mycobacterial infection (TB or NTM)	3.2–10.9	–	AATD (62.8)[h], idiopathic (44.9), CVID (42.9) and PCD (25.0)	3
M. tuberculosis[u]	1.0–5.3<br>[1.0–3.9]	28.4	Post-TB (5.7), idiopathic (0.1), post-infectious (other than TB; 0.1), COPD (0.0), asthma (0.0), CTD (0.0), immunodeficiency (0.0), GERD (0.0)[o]	5
Non-tuberculosis mycobacteria[v]	0.4–44.5<br>[0.4–4.1]	–	Post-TB (26.1), other aetiologies (4.9)[f]	20
			COPD (0.6)[i], other aetiologies (1.3)	
			COPD (0.0–6.0)[j]	
			AATD (5.2)[l]	
			Post-TB (7.2), GERD (4.1), idiopathic (4.0), asthma (3.9), immunodeficiency (2.9), CTD (2.8), COPD (2.2), post-infectious (other than TB; 1.9)[o]	
**Fungal infections**				
Fungal infection[w]	1.2–33.1<br>[1.2–33.1]	–	CTD (1.7), post-TB (1.6), post-infectious (other than TB; 1.3), idiopathic (1.2), GERD (1.0), COPD (1.0), asthma (0.8), immunodeficiency (0.0)[o]	4
Aspergillus species	0.7–38.9<br>[6.3–19.4]	–	Post-TB (0.0), other aetiologies (1.0)[f]	10
			COPD (2.5)[i], other aetiologies (3.3)	
			COPD (4.0–6.0)[j]	
Aspergillus fumigatus	0.0–25.5<br>[0.3–25.5]	–	PCD (9.3)[x], other aetiologies (4.3)	9

Square brackets indicate data from larger or multicentre studies only. Blank cells indicate infections for which data were not reported in children. All data from individual studies are available in the supplementary Excel file.

[a]The maximum value reported here was from a study in which participation required airway *P. aeruginosa* isolation in the past and at screening [28].

[b]Studies reporting in patients with idiopathic or post-infectious bronchiectasis; no comparison with other aetiologies [3, 10, 16].

[c]Significantly more children with CF-related bronchiectasis had *P. aeruginosa* infection compared with children with NCFBE ($p<0.001$) [29].

[d]Studies reporting in patients with CF-related bronchiectasis (the maximum value is reported in patients on long-term ventilation); no comparison with other aetiologies [17, 30].

[e]Significantly more patients with idiopathic bronchiectasis had *P. aeruginosa* infection compared with post-TB and other aetiologies ($p<0.05$ for both comparisons) [1].

[f]NTM was isolated in significantly more patients (hospitalised for an exacerbation) with post-TB bronchiectasis compared with patients with other aetiologies (p<0.001); however, no significant differences between post-TB bronchiectasis and other aetiologies were reported for *P. aeruginosa*, *H. influenzae* or aspergillus species isolation (p=0.59, p=1.0 and p=1.0, respectively) [7].

[g]In one study, *P. aeruginosa* infection was more prevalent in patients with PCD-related bronchiectasis compared with other aetiologies [19] while in a second study the opposite was true [18]; however, no statistical analyses were carried out in either study.

[h]There were no significant differences in *S. aureus* infection between aetiologies (no p-value reported) [5]; however, the percentage of patients with a positive *P. aeruginosa* culture was significantly higher in patients with PCD-related bronchiectasis compared with other aetiologies (p<0.001), and the percentage of patients with a positive mycobacterial culture was significantly higher in AATD-related bronchiectasis compared with other aetiologies (p=0.001) [5].

[i]No significant difference between COPD-related bronchiectasis and other aetiologies in the percentage of patients with *P. aeruginosa*, *H. influenzae*, *S. aureus*, *S. pneumoniae*, *M. catarrhalis*, *M. tuberculosis* or aspergillus species infection was found (no p-values given) [21].

[j]Study reported in patients with COPD-related bronchiectasis of different severities; no comparison with other aetiologies [22].

[k]Significantly more patients with COPD-related bronchiectasis had *P. aeruginosa* infection compared with idiopathic and RA-associated bronchiectasis (p<0.05 for both comparisons) [23].

[l]Study reporting in patients with AATD-related bronchiectasis; no comparison with other aetiologies [20].

[m]Significantly fewer patients with IBD-related bronchiectasis had *P. aeruginosa* infection compared with other aetiologies (p=0.009) [31].

[n]Significant difference between aetiologies reported (p<0.001; comparison of all aetiologies, no head-to-head comparisons performed) [25].

[o]There were no significant differences between aetiologies for *H. parainfluenzae*, *S. aureus* or fungal isolation (p=0.089, p=0.086 and p=0.787, respectively); however, significant differences between aetiologies were reported for *P. aeruginosa*, *H. influenzae*, *M. tuberculosis* and NTM isolation (p=0.002, p=0.001, p<0.001 and p<0.001, respectively) [32].

[p]Significant differences between patients with COPD-related, genetic or congenital and idiopathic bronchiectasis (p=0.0276) [26].

[q]No statistical analyses performed in any of the studies [6, 33]. ‘Other’ includes RA, PCD, GERD and NTM infection.

[r]In both studies, *H. influenzae* and *S. pneumoniae* infection was more prevalent and *S. aureus* infection was less prevalent in patients with PCD-related bronchiectasis compared with other aetiologies; however, no statistical analyses were carried out in either study [18, 19].

[s]No statistical analyses performed in any of the studies [6, 26, 33]. ‘Other’ includes RA, PCD, GERD and NTM infection.

[t]Additional data on methicillin-sensitive and methicillin-resistant *S. aureus* infection and chronic infection can be seen in the online supplement.

[u]It is possible that the prevalence in many studies is zero but is not reported.

[v]Further detail on specific NTM species including *M. avium*, *M. abscessus*, *M. chelonae*, *M. kansasii* and *M. gordonae* can be seen in the supplementary Excel file.

[w]The most frequently reported fungal infection was by aspergillus species; other fungal infections can be seen in the supplementary Excel file.

[x]*Aspergillus fumigatus* infection was more prevalent in patients with PCD-related bronchiectasis compared with other aetiologies; however, no statistical analyses were carried out [19].

AATD, alpha-1 antitrypsin deficiency; ABPA, allergic bronchopulmonary aspergillosis; CF, cystic fibrosis; COPD, chronic obstructive pulmonary disease; CTD, connective tissue disease; CVID, common variable immunodeficiency; GERD, gastro-oesophageal reflux disease; IBD, inflammatory bowel disease; NCFBE, non-cystic fibrosis bronchiectasis; NTM, non-tuberculous mycobacteria; PCD, primary ciliary dyskinesia; PID, primary immunodeficiency; RA, rheumatoid arthritis; TB, tuberculosis.

Supplementary Table S5. Most commonly reported comorbidities in patients with bronchiectasis overall and in individual bronchiectasis aetiologies

Comorbidity	Prevalence overall	Prevalence by aetiology	Number of studies
**Cardiovascular comorbidities**			
**Cardiovascular disease (including ischaemic heart disease)**	3.3–65.5 [4.3–50.0]	COPD (5.6), post-infectious (other than TB; 5.0), post-TB (4.8), GERD (4.5), asthma (3.7), CTD (3.5), idiopathic (3.3), immunodeficiency (2.9)[a]	29
		Idiopathic or post-infectious (3.0–4.8)[b]	
		Post-TB (10.9), other aetiologies (10.8)[c]	
		Asthma (37.0), COPD (31.0), other aetiologies (23.6), idiopathic (19.6), post-infectious (13.0), ABPA (12.8), post-TB (12.4)[d]	
**Heart failure**	0.0–34.0 [2.1–34.0]	–	13
**Cerebrovascular disease (including stroke)**	0.0–27.2 [0.0–27.2]	Post-infectious (other than TB; 5.7), post-TB (4.7), COPD (4.3), GERD (3.3), CTD (3.3), idiopathic (3.3), asthma (2.1), immunodeficiency (1.5)[a]	18
		Post-TB (0.8), post-infectious (0.6), idiopathic (0.0), ABPA (0.0), COPD (0.0), asthma (0.0), other aetiologies (0.0)[d]	
**Hypertension**	1.3–54.3 [12.1–54.3]	COPD (16.0), autoimmune (14.0), idiopathic (7.0), immunodeficiency (7.0), post-infectious (3.0), asthma (0.0), tumour (0.0), genetic/congenital (0.0)[d]	21
**Respiratory comorbidities**			
**Asthma**	1.1–68.4 [5.8–32.9]	Post-TB (10.9), other aetiologies (19.6)[c]	43
		ABPA (80.6), idiopathic (19.4), post-infectious (16.5), other aetiologies (13.5), post-TB (10.1), COPD (6.9)[d]	
**COPD**	0.0–86.5 [9.0–83.7]	Idiopathic or post-infectious (19.2)[b]	44
		Post-TB (26.1), other aetiologies (20.6)[c]	
		Post-TB (27.3), post-infectious (17.3), other aetiologies (16.9), idiopathic (16.0), ABPA (5.6), asthma (0.0)[d]	
**Sinusitis**	5.9–37.5 [8.3–10.1]	COPD (7.2), other aetiologies (26.5)[e]	8
		Idiopathic (22.2), immunodeficiency (14.3), asthma (10.0), genetic/congenital (6.7), post-infectious (6.5), COPD (0.0), tumour (0.0), autoimmune (0.0)[d]	

**Metabolic comorbidities**			
**Diabetes**	0.0–27.7<br>[0.0–27.7]	Post-infectious (other than TB; 9.3), COPD (8.0), post-TB (7.3), asthma (5.3), idiopathic (5.1), immunodeficiency (4.4), CTD (4.4), GERD (4.3)[a]<br>Idiopathic or post-infectious (9.5)[b]<br>Post-TB (8.7), other aetiologies (14.7)[c]<br>COPD (31.9), asthma (16.7), post-TB (15.6), idiopathic (14.3), post-infectious (12.4), other aetiologies (9.0), ABPA (5.6)[d]	41
**Dyslipidaemia (including hyperlipidaemia and hypercholesterolaemia)**	9.3–45.3<br>[9.3–43.0]	–	10
**Malignancy-related comorbidities**			
**Cancer/neoplasm/malignancy/tumour**	1.0–30.2<br>[5.8–15.5]	Idiopathic or post-infectious (3.0)[b]	12
**Haematological malignancy (including leukaemia)**	0.0–6.5<br>[0.4–4.2]	Post-infectious (other than TB; 5.5), immunodeficiency (4.4), post-TB (4.4), idiopathic (4.0), CTD (3.8), COPD (3.7), asthma (2.8), GERD (2.0)[a]	5
**Solid tumour**	0.8–38.9<br>[0.8–12.0]	Post-infectious (other than TB; 9.5), post-TB (9.4), COPD (6.4), idiopathic (6.2), CTD (4.9), asthma (4.0), immunodeficiency (3.9), GERD (3.8)[a]<br>COPD (1.7), other aetiologies (1.1), post-TB (0.9), post-infectious (0.8), idiopathic (0.6), ABPA (0.0), asthma (0.0)[d]	4
**Lung cancer/lung malignancy only**	0.7–3.7<br>[1.8–3.7]	Post-TB (0.0), other aetiologies (1.0)[c]	3
**Bone and joint-related comorbidities**			
**Osteoporosis**	5.9–20.6<br>[5.9–17.7]	Idiopathic or post-infectious (19.0)[b]<br>Asthma (13.0), COPD (11.2), post-TB (7.2), other aetiologies (6.7), post-infectious (5.9), ABPA (5.6), idiopathic (1.7)[d]	11
**Rheumatological disease (including rheumatoid arthritis)**	0.0–16.1<br>[2.7–12.3]	–	8
**Neurological comorbidities**			
**Neurological disease**	0.0–12.0<br>[2.6–8.5]	Post-TB (2.2), other aetiologies (8.8)[c]	6
**Anxiety**	17.3–39.9	–	11
**Depression**	4.3–30.1<br>[4.3–15.7]	–	6

**Renal and hepatic comorbidities**			
**Chronic renal disease/failure/ impairment**	0.0–16.3 [1.2–15.8]	Post-TB (3.9), COPD (3.4), CTD (3.3), post-infectious (other than TB; 3.3), immunodeficiency (2.4), asthma (2.4), idiopathic (1.8), GERD (1.5)[a]	16
		COPD (2.6), post-TB (1.2), other aetiologies (1.1), post-infectious (1.0), ABPA (1.0), idiopathic (0.9), asthma (0.0)[d]	
**Liver cirrhosis/disease**	0.0–13.9 [1.6–6.4]	Immunodeficiency (7.8), post-TB (4.9), COPD (4.6), CTD (4.6), post-infectious (other than TB; 3.9), idiopathic (3.7), asthma (3.2), GERD (2.3)[f]	10
		Post-TB (2.2), other aetiologies (0.0)[c]	
**Gastrointestinal comorbidities**			
**GERD**	14.7–54.0 [15.2–47.0]	Post-TB (2.2), other aetiologies (2.9)[c]	16
		Other aetiologies (21.3), post-infectious (20.4), post-TB (17.9), COPD (17.2), idiopathic (9.6), ABPA (9.2), asthma (7.4)[d]	

Square brackets indicate data from larger or multicentre studies only. Blank cells indicate comorbidities for which data were not reported in individual aetiologies. All data from individual studies are available in the supplementary Excel file.

[a]Significant differences between aetiologies reported (p<0.001) [32].

[b]Studies reporting in patients with idiopathic or post-infectious bronchiectasis; no comparison with other aetiologies [8, 13, 16].

[c]No significant difference in the prevalence of comorbid ischaemic heart disease (p=0.99), asthma (p=0.19), COPD (p=0.46), diabetes (p=0.53), lung cancer (p=1.00), neurological disease (p=0.44), liver disease (p=0.31) or GERD (p=0.31) between patients (hospitalised for an exacerbation) with post-TB bronchiectasis and other aetiologies [7].

[d]No statistical analyses were performed in these studies [6, 13, 26]. 'Other' includes RA, PCD, GERD and NTM infection.

[e]Significantly fewer patients with COPD-related bronchiectasis had sinusitis compared with other aetiologies (p<0.001) [21].

[f]Significant differences between aetiologies reported (p=0.003) [32].

ABPA, allergic bronchopulmonary aspergillosis; COPD, chronic obstructive pulmonary disease; CTD, connective tissue disease; GERD, gastro-oesophageal reflux disease; NTM, non-tuberculosis mycobacteria; PCD, primary ciliary dyskinesia; RA, rheumatoid arthritis; TB, tuberculosis.

Supplementary Table S6. Exercise capacity and physical activity, sedentary time and steps per day in patients with bronchiectasis overall, in children with bronchiectasis and in individual bronchiectasis aetiologies

	Description	Range of means/ medians overall	Range of means/ medians in children	Means/ medians by aetiology	Number of studies
	**Walk tests (m)**				
6MWT	Internally paced (patient determines how fast they walk) sub-maximal test. In healthy individuals, the 6MWT distance ranges from 400–700 m	252–578[a] [421–578][a] 434–515[b]	–	Idiopathic or post-infectious (439)[c]	18
ISWT	Externally paced (patient is required to pace their activity in time with a signal) maximal test. In healthy individuals, the ISWT distance ranges from 600–800 m	238–608[a] [464–474][a] 190–390[b]	–	–	8
	**Physical activity (minutes per day)**				
Light		186–341.6[a]	260.9	–	4
Moderate		81.9–127.3[a]	–	–	3
Moderate-to-vigorous		82.4–125.3[a]	48.6	–	2
Vigorous		0.6–9.9[a]	–	–	3
	**Steps per day**				
Steps		4740–7563[b]	8230	–	4
	**Sedentary time (hours per day)**				
Sedentary time		6.8–16.4[a]	7.0	–	5

Square brackets indicate data from larger or multicentre studies only. Blank cells indicate measures of exercise capacity for which data were not reported in children or in individual aetiologies. All data from individual studies are available in the supplementary Excel file.

[a]Studies reporting mean.

[b]Studies reporting median.

[c]Study reporting in patients with idiopathic or post-infectious bronchiectasis; no comparison with other aetiologies [8].

6MWT, Six-Minute Walk Test; ISWT, Incremental Shuttle Walk Test.

Supplementary Table S7. Most commonly reported respiratory medications in patients with bronchiectasis overall and in individual bronchiectasis aetiologies

Treatment	Prevalence	Prevalence by aetiology	Number of studies
**Antibiotics – general**			
Inhaled/nebulised antibiotics at baseline	0.0–58.0 [0.0–44.9]	Idiopathic or post-infectious (4.8)[a]	15
		PCD (39.6), COPD (14.3), idiopathic (11.1), systemic diseases (10.3), post-infectious (9.7), PID (7.4), asthma (4.8)[b]	
		ABPA (9.7), COPD (7.8), asthma (5.6), other aetiologies (4.5), post-infectious (4.1), idiopathic (2.1), post-TB (1.8)[c]	
Long-term/chronic/maintenance inhaled antibiotics	0.7–36.0 [0.7–36.0]	–	13
Long-term oral antibiotics	9.0–58.0 [9.0–19.4]	–	6
**Antibiotics – macrolides**			
Macrolides in previous 6 months	40.8–44.0	–	4
Macrolides at baseline	12.0–69.0 [20.2–30.8]	Idiopathic or post-infectious (57.1)[a]	11
Habitual/chronic/long-term macrolides	4.3–83.0 [4.3–54.0]	Idiopathic or post-infectious (7.7)[a]	22
		Post-infectious (9.6), other aetiologies (7.9), COPD (6.9), post-TB (6.5), asthma (3.7), idiopathic (3.2), ABPA (2.0)[c]	
**Corticosteroids**			
ICS in previous 6 months	22.0–26.5	–	3
ICS at baseline	3.8–88.4 [3.8–88.4]	COPD (82.7), other aetiologies (65.3)[d]	40
		Idiopathic or post-infectious (13.6–52.4)[a]	
Habitual/chronic/long-term/maintenance ICS	3.2–80.4 [3.2–80.4]	–	7
LABA + ICS at baseline	34.3–88.5 [34.3–88.5]	Idiopathic or post-infectious (31.3–69.1)[a]	6
LAMA + LABA + ICS at baseline	16.7–27.6 [16.7–27.6]	–	1
Oral corticosteroid at baseline	0.0–25.6 [9.0–13.0]	–	8
Habitual/chronic/long-term oral corticosteroid	2.0–13.3 [3.8–8.5]	–	6
**Bronchodilators**			
Any bronchodilator at baseline	45.0–95.0 [45.0–83.2]	COPD (94.0), other aetiologies (73.5)[d]	10
SABA at baseline	22.5–89.3 [22.5–59.1]	Idiopathic or post-infections (18.5–25.0)[a]	7
LABA at baseline	0.0–88.7 [6.4–88.7]	–	14
Habitual/chronic LABA	3.2–81.4 [3.2–81.4]	–	2
LAMA at baseline	0.0–80.0 [25.7–65.3]	Idiopathic or post-infections (6.3–40.9)[a]	9
Habitual/chronic LAMA	12.8–61.7	–	1

	[12.8–61.7]		
**Mucolytics**			
Mucolytic in previous 6 months	72.0–77.6	–	4
Mucolytic at baseline	1.4–58.8<br>[37.8–49.6]	–	8
Hypertonic saline at baseline	1.3–73.1<br>[6.8]	–	4
**Oxygen**			
Long-term/habitual oxygen therapy	0.0–19.8<br>[0.0–19.8]	–	10

Square brackets indicate data from larger or multicentre studies only. Blank cells indicate treatments for which data were not reported in individual aetiologies. All data from individual studies are available in the supplementary Excel file.

[a]Studies reporting in patients with idiopathic or post-infectious bronchiectasis; no comparison with other aetiologies [3, 8, 11, 12].

[b]Significant difference between aetiologies (p<0.001; comparison of all aetiologies, no head-to-head comparisons performed) [25].

[c]No statistical analyses performed [6]. ‘Other’ includes RA, PCD, GERD and NTM infection.

[d]A significantly greater proportion of patients with COPD-related bronchiectasis were treated with ICS and any bronchodilator compared with other aetiologies (p<0.001 for both comparisons) [21].

ABPA, allergic bronchopulmonary aspergillosis; COPD, chronic obstructive pulmonary disease; GERD, gastro-oesophageal reflux disease; ICS, inhaled corticosteroids; LABA, long-acting beta agonists; LAMA, long-acting muscarinic antagonists; NTM, non-tuberculosis mycobacteria; PCD, primary ciliary dyskinesia; PID, primary immunodeficiency; RA, rheumatoid arthritis; SABA, short-acting beta agonists; TB, tuberculosis.

References

1. Al-Harbi A, Al-Ghamdi M, Khan M, et al. Performance of multidimensional severity scoring systems in patients with post-tuberculosis bronchiectasis. *Int J Chron Obstruct Pulmon Dis* 2020; 15: 2157-2165.
2. Qi Q, Wang W, Li T, et al. Aetiology and clinical characteristics of patients with bronchiectasis in a Chinese Han population: A prospective study. *Respirology* 2015; 20: 917-924.
3. Qi Q, Ailiyaer Y, Liu R, et al. Effect of N-acetylcysteine on exacerbations of bronchiectasis (BENE): a randomized controlled trial. *Respir Res* 2019; 20: 73.
4. Mitchelmore P, Polverino E, Rademacher J, et al. Immunodeficiency associated bronchiectasis in the European Bronchiectasis Registry (EMBARC). *Eur Respir J* 2020; 56: 2057.
5. Eden E, Choate R, Barker A, et al. The clinical features of bronchiectasis associated with Alpha-1 antitrypsin deficiency, common variable immunodeficiency and primary ciliary dyskinesia--results from the U.S. Bronchiectasis Research Registry. *Chronic Obstr Pulm Dis* 2019; 6: 145-153.
6. Dhar R, Singh S, Talwar D, et al. Bronchiectasis in India: results from the European Multicentre Bronchiectasis Audit and Research Collaboration (EMBARC) and Respiratory Research Network of India Registry. *Lancet Glob Health* 2019; 7: e1269-e1279.
7. Fong I, Low TB, Yii A. Characterisation of the post-tuberculous phenotype of bronchiectasis: A real-world observational study. *Chron Respir Dis* 2022; 19: 14799731221098714.
8. Artaraz A, Crichton ML, Finch S, et al. Development and initial validation of the bronchiectasis exacerbation and symptom tool (BEST). *Respir Res* 2020; 21: 18.
9. Lee E, Shim JY, Kim HY, et al. Clinical characteristics and etiologies of bronchiectasis in Korean children: A multicenter retrospective study. *Respir Med* 2019; 150: 8-14.
10. Wilson R, Welte T, Polverino E, et al. Ciprofloxacin dry powder for inhalation in non-cystic fibrosis bronchiectasis: a phase II randomised study. *Eur Respir J* 2013; 41: 1107-1115.
11. Stockley R, De Soyza A, Gunawardena K, et al. Phase II study of a neutrophil elastase inhibitor (AZD9668) in patients with bronchiectasis. *Respir Med* 2013; 107: 524-533.
12. Aksamit T, De Soyza A, Bandel TJ, et al. RESPIRE 2: a phase III placebo-controlled randomised trial of ciprofloxacin dry powder for inhalation in non-cystic fibrosis bronchiectasis. *Eur Respir J* 2018; 51: 1702053.
13. Soyza AD, Pavord I, Elborn JS, et al. A randomised, placebo-controlled study of the CXCR2 antagonist AZD5069 in bronchiectasis. *Eur Respir J* 2015; 46: 1021-1032.
14. Ailiyaer Y, Wang X, Zhang Y, et al. A prospective trial of nebulized amikacin in the treatment of bronchiectasis exacerbation. *Respiration* 2018; 95: 327-333.
15. Watz H, Nagelschmitz J, Kirsten A, et al. Safety and efficacy of the human neutrophil elastase inhibitor BAY 85-8501 for the treatment of non-cystic fibrosis bronchiectasis: A randomized controlled trial. *Pulm Pharmacol Ther* 2019; 56: 86-93.
16. Bedi P, Chalmers JD, Goeminne PC, et al. The BRICS (Bronchiectasis Radiologically Indexed CT Score): A Multicenter Study Score for Use in Idiopathic and Postinfective Bronchiectasis. *Chest* 2018; 153: 1177-1186.
17. Muñoz G, de Gracia J, Giron R, et al. Validation of a Spanish version of the Leicester Cough Questionnaire in cystic fibrosis. *Chron Respir Dis* 2021; 18: 14799731211036903.
18. Paris D, Palomba L, Mirra V, et al. NMR profiling of exhaled breath condensate defines different metabolic phenotypes of non-cystic fibrosis bronchiectasis. *Int J Mol Sci* 2020; 21: 8600.
19. Dettmer S, Ringshausen F, Vogel-Claussen J, et al. Computed tomography in adult patients with primary ciliary dyskinesia: Typical imaging findings. *PLOS ONE* 2018; 13: e0191457.
20. Loebinger M, Aliberti S, Menendez R, et al. Alpha-1 antitrypsin deficiency in patients with bronchiectasis: data from the European Bronchiectasis Registry EMBARC. *Eur Respir J* 2020; 56: 3330.
21. de la Rosa D, Martínez-Garcia M-A, Giron RM, et al. Clinical impact of chronic obstructive pulmonary disease on non-cystic fibrosis bronchiectasis. A study on 1,790 patients from the Spanish Bronchiectasis Historical Registry. *PLOS ONE* 2017; 12: e0177931.
22. Gatheral T, Kumar N, Sansom B, et al. COPD-related bronchiectasis; independent impact on disease course and outcomes. *COPD* 2014; 11: 605-614.
23. De Soyza A, McDonnell MJ, Goeminne PC, et al. Bronchiectasis rheumatoid overlap syndrome is an independent risk factor for mortality in patients with bronchiectasis: A multicenter cohort study. *Chest* 2017; 151: 1247-1254.
24. Masekela R, Anderson R, Gongxeka H, et al. Lack of efficacy of an immunomodulatory macrolide in childhood HIV related bronchiectasis: a randomised, placebo-controlled trial. *JAA* 2013; 5: 044-049.
25. Olveira C, Padilla A, Martínez-García M-Á, et al. Etiology of bronchiectasis in a cohort of 2047 patients. An analysis of the Spanish Historical Bronchiectasis Registry. *Arch Bronconeumol (Engl Ed)* 2017; 53: 366-374.

26. Buscot M, Pottier H, Marquette CH, et al. Phenotyping adults with non-cystic fibrosis bronchiectasis: A 10-year cohort study in a French Regional University Hospital Center. *Respiration* 2016; 92: 1-8.
27. Navas-Bueno B, Casas-Maldonado F, Padilla-Galo A, et al. High adherence, microbiological control and reduced exacerbations in patients with non-cystic fibrosis bronchiectasis treated with nebulised colistin. A prospective observational study. *Arch Bronconeumol* 2022; 58: 834-836.
28. VanDevanter DR, Gonda I, Dahms J, et al. Microbiological changes observed over 48 weeks of treatment with inhaled liposomal ciprofloxacin in individuals with non-cystic fibrosis bronchiectasis and chronic Pseudomonas aeruginosa lung infection. *Clin Microbiol Infect* 2019; 25: 1532-1538.
29. El Basha N. Impact of underlying cause of bronchiectasis on clinical outcome: A comparative study on CF and Non-CF bronchiectasis in Egyptian children. *Egypt Pediatr Assoc Gazette* 2018; 66: 49-53.
30. Sobala R, Carlin H, Fretwell T, et al. An observational study of Pseudomonas aeruginosa in adult long-term ventilation. *ERJ Open Research* 2022; 8: 00687-02021.
31. Soyza AD, Chalmers J, Dimakou K, et al. Impact of inflammatory bowel disease in bronchiectasis (IBD-BR) data from the EMBARC registry. *Eur Respir J* 2018; 52: PA2678.
32. Huang HY, Chung FT, Lo CY, et al. Etiology and characteristics of patients with bronchiectasis in Taiwan: a cohort study from 2002 to 2016. *BMC Pulm Med* 2020; 20: 45.
33. Sharif N, Baig MS, Sharif S, et al. Etiology, clinical, radiological, and microbiological profile of patients with non-cystic fibrosis bronchiectasis at a tertiary care hospital of Pakistan. *Cureus* 2020; 12: e7208.

Figure legends

Supplementary Figure S1. PRISMA study identification flow chart

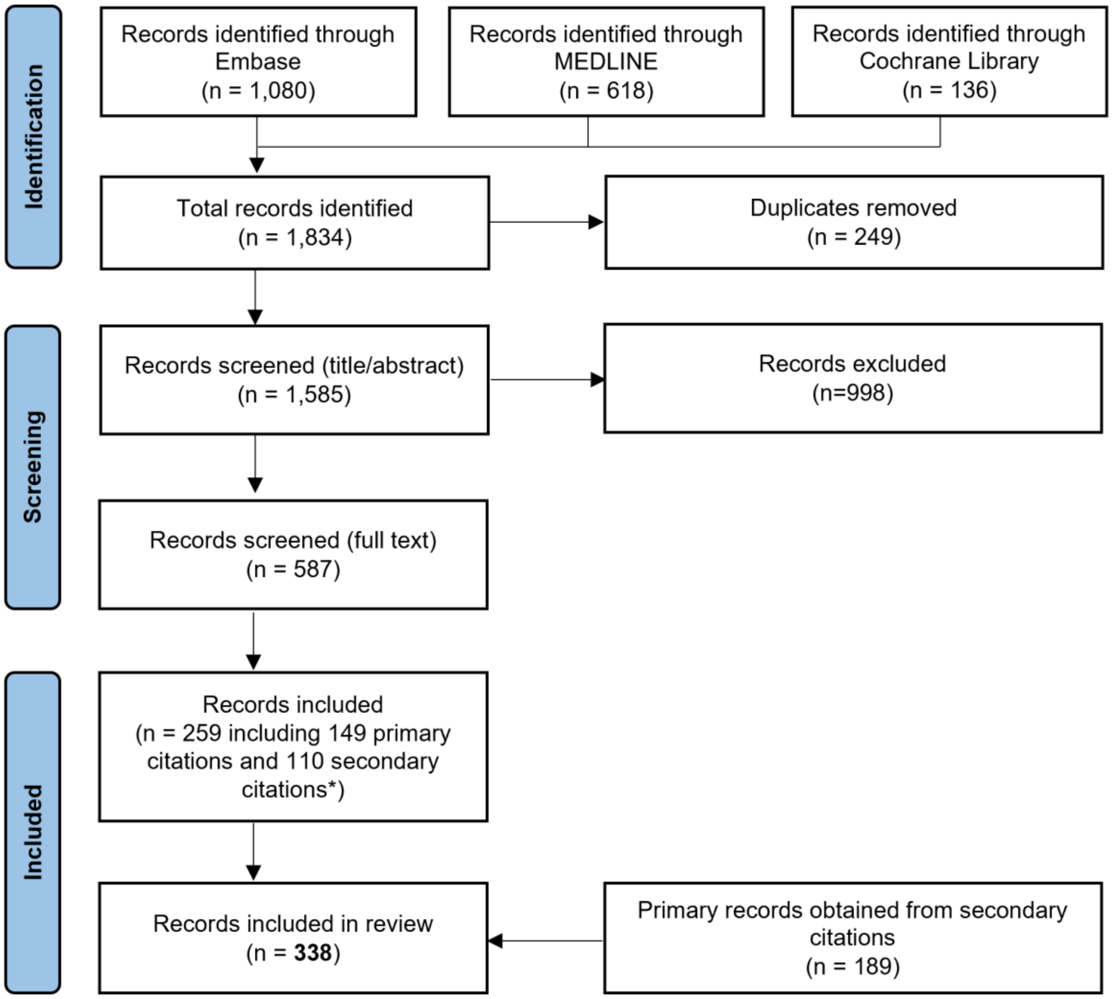

*Including literature reviews, systematic reviews and meta-analyses, editorials and letters to the editor.

Supplementary Figure S2. Most frequently reported aetiologies (range of %) (a) overall and (b) in children

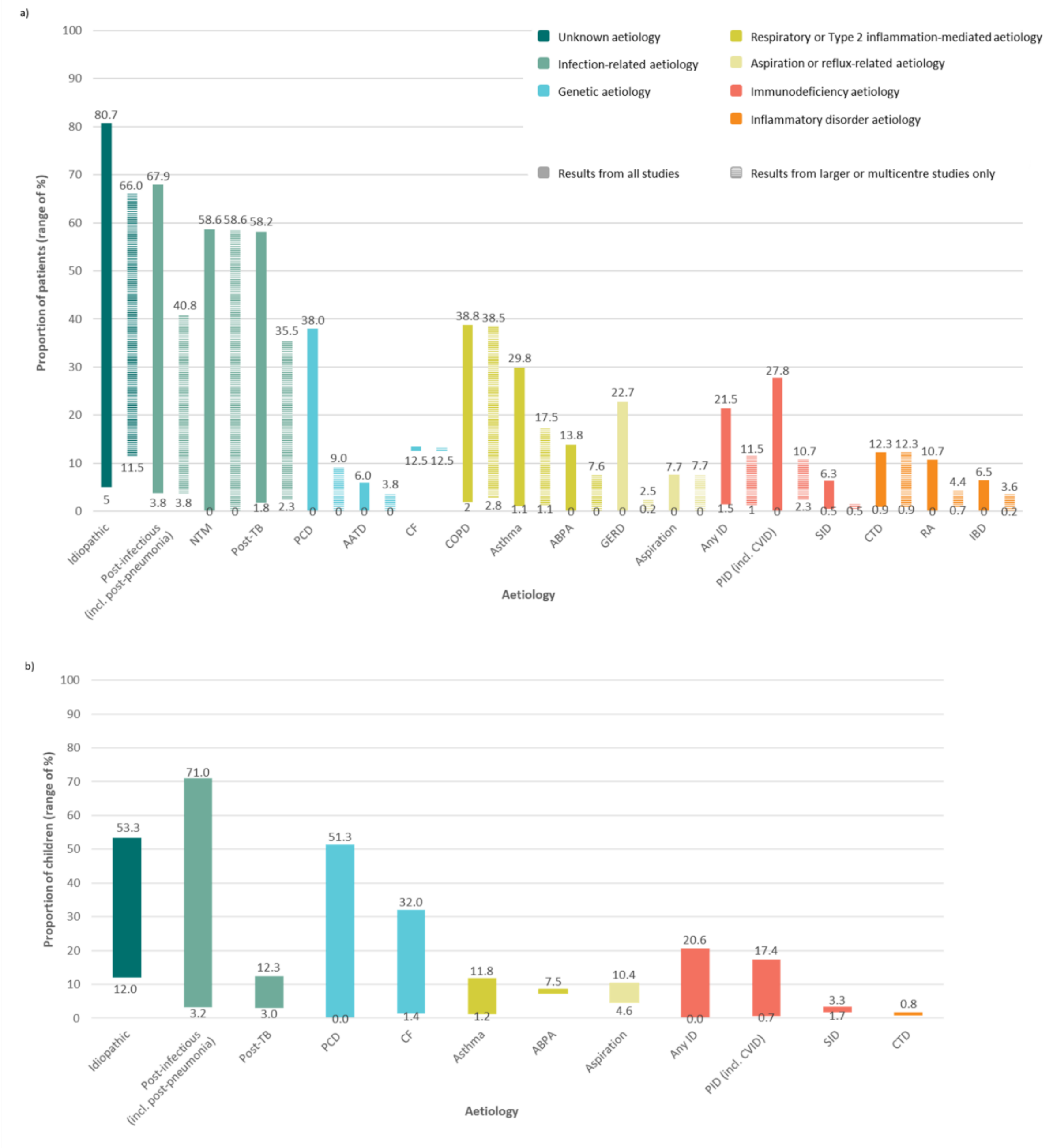

AATD, alpha-1 antitrypsin deficiency; ABPA, allergic bronchopulmonary aspergillosis; CF, cystic fibrosis; COPD, chronic obstructive pulmonary disease; CTD, connective tissue disease; CVID, common variable immunodeficiency; GERD, gastro-oesophageal reflux disease; IBD, inflammatory bowel disease; ID, immunodeficiency; NTM, non-tuberculosis mycobacteria; PCD, primary ciliary dyskinesia; PID, primary immunodeficiency; RA, rheumatoid arthritis; SID, secondary immunodeficiency; TB, tuberculosis.
